# Biomimetic artificial organelles with in vitro and in vivo activity triggered by reduction in microenvironment

**DOI:** 10.1038/s41467-018-03560-x

**Published:** 2018-03-19

**Authors:** T. Einfalt, D. Witzigmann, C. Edlinger, S. Sieber, R. Goers, A. Najer, M. Spulber, O. Onaca-Fischer, J. Huwyler, C. G. Palivan

**Affiliations:** 1 Department of Chemistry, University of Basel, Klingelbergstrasse 80, CH-4056 Basel, Switzerland; 2 Department of Pharmaceutical Sciences, Division of Pharmaceutical Technology, University of Basel, Klingelbergstrasse 50, CH-4056 Basel, Switzerland; 3 Department of Biosystems Science and Engineering, ETH Zürich, Mattenstrasse 26, CH-4058 Basel, Switzerland

## Abstract

Despite tremendous efforts to develop stimuli-responsive enzyme delivery systems, their efficacy has been mostly limited to in vitro applications. Here we introduce, by using an approach of combining biomolecules with artificial compartments, a biomimetic strategy to create artificial organelles (AOs) as cellular implants, with endogenous stimuli-triggered enzymatic activity. AOs are produced by inserting protein gates in the membrane of polymersomes containing horseradish peroxidase enzymes selected as a model for natures own enzymes involved in the redox homoeostasis. The inserted protein gates are engineered by attaching molecular caps to genetically modified channel porins in order to induce redox-responsive control of the molecular flow through the membrane. AOs preserve their structure and are activated by intracellular glutathione levels in vitro. Importantly, our biomimetic AOs are functional in vivo in zebrafish embryos, which demonstrates the feasibility of using AOs as cellular implants in living organisms. This opens new perspectives for patient-oriented protein therapy.

## Abstract (web summary)

The efficacy of stimuli-responsive enzyme delivery systems is usually limited to in vitro applications. Here the authors form artificial organelles by inserting stimuli-responsive protein gates in membranes of polymersomes loaded with enzymes and obtain a triggered functionality both in vitro and in vivo.

## Introduction

Mimicking biological processes by engineering biomimetic nanostructures represents an elegant strategy for addressing problems in various scientific fields, including materials science, chemistry, electronics and medicine^1–3^. By applying a bottom-up biomimetic design (i.e. arranging molecules at the nanoscale via self-assembly), it is possible to combine individual biological units, known for their sophisticated structure and activity (e.g. proteins, lipids, DNA), with robust synthetic materials (e.g. polymers, porous silica surfaces, nanoparticles). This serves to develop nanoscale biomimics with enhanced properties and functionalities^2,4–8^ with potential for a wide range of applications (sensitive biosensing, patient tailored therapeutics, detoxification of environmental pollutants, etc.)^6,9–12^.

Of particular interest are two different concepts that are currently the main focus in this research field: (i) artificial organelles (AOs) based on an essential need to offer efficient solutions for improved therapy and diagnostics^13^ and (ii) protocell systems intended to provide simple models of cells for understanding various internal processes^2,14^. These concepts are complementary, one is essential for advancing medical applications (AOs) whereas the second concept mimics cell behaviour based on very simple systems (protocells). Similarly, as in nature, the sizes of the compartments are completely different: while AOs have nanometre range sizes, protocells reach the micrometre range. Even though protocells represent the first archetypes of an artificial cell, they still inherently lack the material variety and responsiveness found in the most basic cellular structures, and have not yet been investigated in vivo to determine whether they preserve their functionality. AOs are particularly attractive nanoscale biomimics because they can provide a required compound/signal, detoxify harmful compounds, or change cellular conditions and reactions. AOs are based on compartmentalisation of active compounds (enzymes, proteins, catalysts, mimics) within artificial nano-assemblies that reach and function in the intracellular environment, and thus serve as simplified mimics of nature’s own organelles. Various examples of systems with potential to act as AOs have been developed based on liposomes, porous silica nanoparticles and polymer compartments (polymersomes) in combination with biomacromolecules^13–16^. However, very few have been evaluated in vitro to assess their in situ cellular functionality^6,15–18^, and to the best of our knowledge, none has been assessed in vivo. In vivo functionality of such AOs is a crucial factor that is necessary to demonstrate that the concept of AOs is feasible in living organisms, and thus AOs can act as cellular implants.

Notably, natural organelles have membranes, since inside cells compartmentalisation is essential to provide confined reaction spaces for complex metabolic reactions. Therefore, an AO should preserve the compartmentalisation as a key factor in mimicking natural organelles. In this respect, polymer compartments, named polymersomes, are ideal candidates for the creation of AOs, because of their hollow spherical structure with a membrane serving as a border for an inner cavity and their greater mechanical stability than lipid-based compartments, i.e. liposomes^19,20^. In addition, the chemical nature of the copolymers provides the possibility of controlling their properties (e.g. size, stability biocompatibility, flexibility, stimuli-responsiveness)^2,21^. Polymersomes have been shown to serve either as carriers for biomolecules and mimics^1,2,21,22^, or more recently for development of nanoreactors and even the generation of AOs^2,6,11,23^. A key factor for supporting in situ reactions^20,24^ is to render the polymersome membranes permeable for substrates and products. An elegant approach bioinspired from the cell membrane is to incorporate biopores and membrane proteins^25–27^. Selective membrane permeability towards protons and ions is achieved by inserting small pore forming peptides^27^, while membrane proteins induce size-dependent cut-off permeability^26,28–30^ or even mediate the diffusion of specific molecules^10,31^. The few reported AOs exhibit enzymatic reactions either inside porous polymersomes^6,16,32^ or inside polymersomes equipped with channel porins^17^, with the aim of emulating cellular pathways (e.g. reactive oxygen species detoxification or glucose oxidation).

Another essential factor for tuning AO functionality is a triggered response to its environment, as, for example, the redox state of the cell, which regulates various processes involved in cellular signalling pathways^33,34^. While there are a few reported examples of polymersomes with a stimuli-responsive permeable membrane based on the incorporation of genetically or chemically modified membrane proteins^35^, only two of them have served for the design of catalytic nanocompartments^36,37^, and none has been used to control reactions inside AOs. Activation of the AO by a specific endogenous stimulus inside cells represents a challenging step in development of functional AOs in vivo. The design of AOs with triggered activity and the demonstration of their in vivo functionality represent necessary steps towards the creation of cell implants, and the provision of smart solutions for personalised medicine by a straightforward change of the biomolecules inside the AOs.

Here, we present a strategy for designing AOs with an in situ enzymatic reaction that is triggered by the presence of an intracellular stimulus, and demonstrate in vitro and in vivo functionality. Genetically modified outer membrane protein F (OmpF) porins were incorporated into polymersomes to induce redox responsiveness to the membrane, and horseradish peroxidase (HRP) simultaneously encapsulated inside their cavity to provide a source of the AO functionality. Such AOs with functionality triggered by intracellular changes represent an advance in mimicking that of nature’s own organelles, especially those that are involved in the redox equilibrium of the cellular homoeostasis. Amphiphilic block copolymers poly(2-methyloxazoline)-*block*-poly(dimethylsiloxane)-*block*-poly(2-methyloxazoline) (PMOXA_*m*_-PDMS_*n*_-PMOXA_*m*_) were used to self-assemble into polymersomes, because such copolymers have already been shown to form membranes in which biopores and membrane proteins can be successfully inserted^36–38^, and to be taken up and to be non-toxic to various cell lines^17^. Once inserted in the polymersome membrane, the modified OmpF porins act as protein gates independent of the insertion direction, i.e. orientation in the membrane^36,37,39,40^, and trigger the in situ HRP enzymatic reaction when a stimulus is present in the cellular environment. HRP was selected as model enzyme, because peroxidases play a significant role in the redox homoeostasis of cells and cell apoptosis^41^. This strategy of providing stimuli-responsiveness to polymersome membranes neither affects the membrane integrity, as for stimuli-responsive synthetic membranes of compartments^42^, nor the size and structure of the polymersomes. Crucial steps were the evaluation of AO toxicity and functionality in human epithelial tumour cells (HeLa cells), and once these were established in vivo tolerability, preservation of the AO structure, and in situ regulation of the activity of the encapsulated enzyme in the vertebrate zebrafish embryo (ZFE) model.

## Results

### Bioengineering protein gates by modification of channel porins

**Fig. 1:**
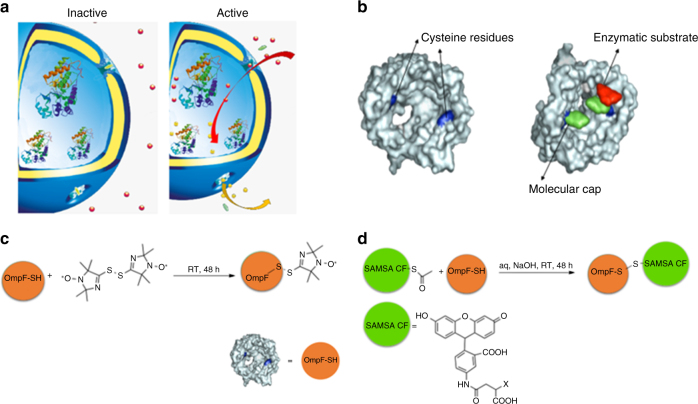
Engineering stimuli-responsive OmpF. **a** Schematic representation of modified OmpF acting as a gate in catalytic nanocompartments. **b** Molecular representation of the OmpF-M cysteine mutant^37^. **c** Chemical modification of OmpF-M cysteine mutant with the spin probe bis-(2,2,5,5-tetramethyl-3-imidazoline-1-oxyl-4-yl) disulphide. **d** Chemical modification of OmpF-M cysteine mutant with the fluorophore SAMSA-CF

The key factors in the design of AOs with activity triggered by changes in environmental conditions are on-demand permeability of the compartment towards enzymatic substrates/products and structural integrity of the polymersome, which mimics that of natural organelles. Therefore, our biomimetic strategy aimed to equip PMOXA_6_-PDMS_44_-PMOXA_6_ polymersome membranes with protein gates that are responsive to changes in glutathione (GSH) concentrations in intracellular environments, while preserving the structure of the nanocompartment (Fig. 1a).

It has been shown very recently that chemical modifications of amino acid residues at key locations of the OmpF porin backbone influence the translocation of substrates through the pore in a pH-responsive manner^36^. Here we go one step further by using a double mutant of OmpF^37^ to attach molecular caps to genetically introduced cysteine residues that serve to block/unblock the OmpF pore upon changes in redox potential, which occur when the system enters the intracellular microenvironment (Fig. 1b). In contrast to polymersomes with membranes containing OmpF genetically modified to release a payload in reductive conditions^35^, our system controls the overall functionality of the AOs. We chose a cysteine double mutant of OmpF (OmpF-M) because cysteine residues, replacing the amino acids K89 and R270, were expected to form reduction-sensitive disulphide bonds with molecules selected to serve as molecular caps. These molecular caps remain attached in mildly oxidising environments and block substrate diffusion through the pore, whereas in the presence of reducing agents, such as intracellular GSH, their cleavage restores normal passage of small molecular weight molecules (<600 Da) through the OmpF pores. This approach mimics pathways of metabolism regulation, where proteins within the membranes of natural cell organelles are irreversibly activated or deactivated on demand^43,44^. In addition, we were interested in developing an irreversible protein gate in order to be able to rapidly evaluate the functionality of the organelle in vivo.

The ability of the cysteine residues of OmpF-M to form disulphide bonds with thiol groups of small molecular weight molecules was examined by two complementary assays, one using a suitable spin probe (bis-(2,2,5,5-tetramethyl-3-imidazoline-1-oxyl-4-yl) disulphide) and the second using the fluorescent dye SAMSA fluorescein (SAMSA-CF) (Fig. 1c, d).

Coupling reaction of the molecular caps with the cysteine residues of OmpF-M resulted in the formation of OmpF conjugates (OmpF-S-S-CF for OmpF conjugated with SAMSA-CF, and OmpF-S-S-NO for OmpF conjugated with bis-(2,2,5,5-tetramethyl-3-imidazoline-1-oxyl-4-yl) disulphide), respectively.

**Fig. 2:**
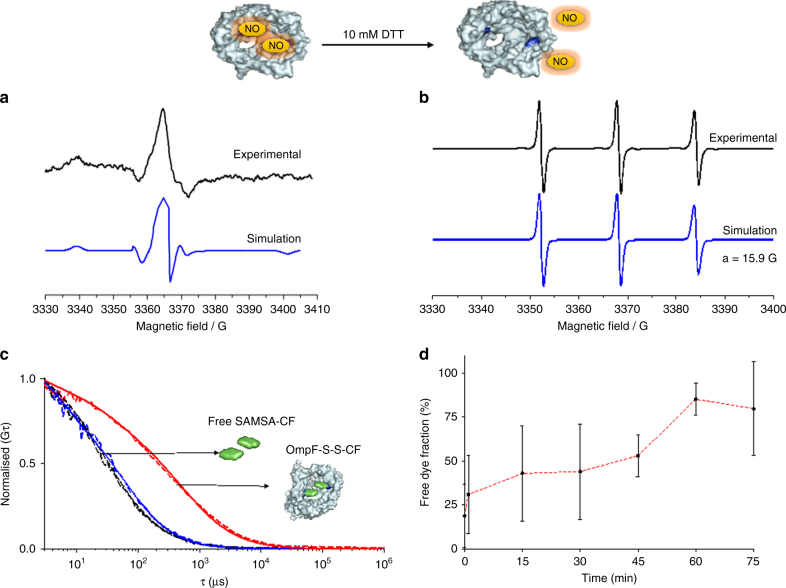
Characterisation of stimuli-responsive OmpF Panel. **a** EPR spectra of bis-(2,2,5,5-tetramethyl-3-imidazoline-1-oxyl-4-yl) disulphide-labelled OmpF-M experimental (black) and simulated (blue) and **b** bis-(2,2,5,5-tetramethyl-3-imidazoline-1-oxyl-4-yl) disulphide-labelled OmpF-M in 1% OG incubated with 10 mM DTT experimental (black) and simulated (blue). **c** Normalised FCS autocorrelation curves for SAMSA-CF in PBS (black), SAMSA-CF in 1% OG (blue) and OmpF-S-S-CF in 1% OG (Red). Dotted line—experimental autocorrelation curves, full line—fit. **d** SAMSA-CF release kinetics from OmpF-M in 30 mM GSH, 1% OG, as measured by FCS and analysed with a two-component fit. Error bars show standard deviations from 60 measurements

Binding of the thiol reactive spin probe to the protein was evaluated by a combination of LC-MS-MS and electron paramagnetic resonance (EPR). Upon in-gel digestion of the porin^45^, LC-MS-MS analysis of the peptide fragments indicated a very high labelling efficiency of the spin probe to cysteine residues of the OmpF-M (96 ± 4%). Standard deviation is based on three measurements. The EPR spectrum of the bis-(2,2,5,5-tetramethyl-3-imidazoline-1-oxyl-4-yl) disulphide in phosphate-buffered saline (PBS) at 298 K consists of an isotropic triplet pattern (Supplementary Figure 1) with a hyperfine coupling *a*_*N*_ value of 15.8 G that is similar to reported values for analogous nitroxide probes where no aggregation was present^46,47^. In contrast, OmpF-S-S-NO gave a broad anisotropic EPR spectrum with no isotropic component, and is similar to that reported for 5-DSA in lipid bilayers or cholesterol aqueous solutions^48^. This EPR spectrum indicates hindered rotation of the nitroxide probe^49^ after binding to the OmpF mutant (OmpF-S-S-NO), and demonstrates successful binding of the bis-(2,2,5,5-tetramethyl-3-imidazoline-1-oxyl-4-yl) disulphide to the modified OmpF mutant (Fig. 2a).

After exposure of OmpF-S-S-NO to 10 mM DTT an isotropic EPR spectrum (*a*_*N*_ value of 15.9 G) characteristic of the freely rotating spin probe was observed (Fig. 2b). This clearly demonstrates that the nitroxide spin probe that is bound to thiol groups of the OmpF-M under oxidative conditions is cleaved in a reductive environment.

SAMSA-CF (Thermo Fischer Scientific) was selected as a molecular cap because its size (molecular weight 521.49 Da) was expected to block the OmpF-M pore, and because of its ability to form cleavable disulphide bonds^50^. Thus, attachment of SAMSA-CF to OmpF-M introduces a stimuli-responsiveness to the pore, and therefore to the polymersome membrane when OmpF-S-S-CF is inserted. In addition, the fluorescent properties of SAMSA-CF allow pore modification to be analysed by a combination of sodium dodecyl sulfate polyacrylamide gel electrophoresis (SDS-PAGE) and fluorescence correlation spectroscopy (FCS).

LC-MS-MS analysis of the peptide fragments indicated a high labelling degree of OmpF-M (81 ± 31%). In addition, a fluorescent band appeared in the SDS-PAGE gel when SAMSA-CF was conjugated to OmpF-M, whereas the OmpF wild type did not interact with the fluorophore; this fluorescent band supports the formation of OmpF-S-S-CF (Supplementary Figure 2). To mimic the intracellular reductive environment, where the GSH concentration is kept at a constantly high level (10 mM GSH) by cytosolic enzymes^51^, such as glutathione reductase, we studied the behaviour of the reduction-responsive molecular caps in a similar environment. Because of the absence of a steady-state concentration and constant regeneration of GSH, we used 30 mM GSH to mimic the intracellular steady state of GSH. In SDS-PAGE the fluorescent band disappeared when the OmpF-S-S-CF was mixed with GSH, indicating successful cleavage of the molecular cap under reductive conditions (Supplementary Figure 2).

The binding of SAMSA-CF to OmpF-M cysteine residues was also evaluated by FCS, because it allows the determination of diffusion coefficients, which are correlated to possible interactions of the fluorescent molecules with supramolecular assemblies, such as polymersomes, liposomes and nanoparticles in the pico- to nanomolar concentration region^9,21,47–52^. We compared the molecular brightness and diffusion times of SAMSA-CF in PBS (pH 7.4), SAMSA-CF in 1% OG PBS (pH 7.4) and SAMSA-CF bound to OmpF (OmpF-S-S-CF) in 1% OG PBS (pH 7.4) (Fig. 2c). A labelling efficiency of an average of two SAMSA-CF molecules per monomer was calculated by comparing the molecular brightness (counts per molecule, CPM in kHz) of SAMSA-CF (2.2 ± 0.7 kHz) with that of protein bound to SAMSA-CF (4.8 ± 0.6 kHz) (Fig. 2c). Standard deviations in molecular brightness are based on individual measurements of the same probe (*n* = 60). In contrast, wild-type OmpF treated similarly to the cysteine mutant OmpF-M, did not present any fluorescence after purification, and there was therefore no binding of SAMSA-CF to OmpF-WT. To determine the kinetics of OmpF pore opening, we used FCS to evaluate the cleavage of SAMSA-CF from labelled OmpF-M upon addition of 30 mM GSH at pH 7.4 by FCS, and analysed the results by a two-component fit. Due to cleavage of the disulphide bonds between the dye and the OmpF-M, the percentage of the free dye increased over time to 85 ± 9%, with a plateau after 1 h (Fig. 2d, Supplementary Figure 3). Standard deviations in the percentage of the free dye are based on individual measurements of the same probe (*n* = 60) during a set time point.

### Catalytic enzyme-polymersome nanocompartments with protein gates

**Fig. 3:**
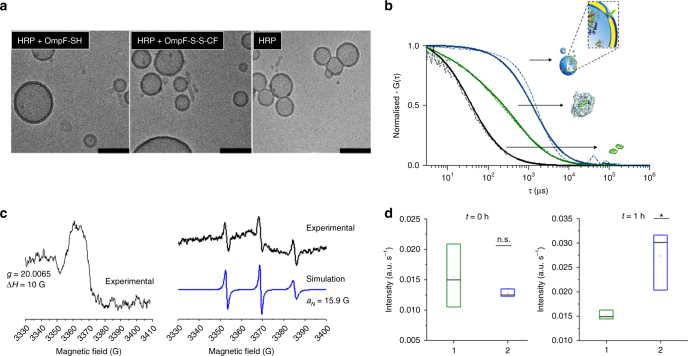
Characterisation of stimuli-responsive catalytic nanocompartments. **a** Cryo-TEM micrographs of: (left) polymersomes loaded with HRP and equipped with OmpF-SH, (middle) polymersomes loaded with HRP and equipped with OmpF-S-S-CF, and (right) polymersomes loaded with HRP without OmpF. Scale bar = 100 nm. **b** Normalised FCS autocorrelation curves of SAMSA-CF in PBS (black) and OmpF-S-S-CF in the membrane of polymersomes (blue). Dotted line = experimental autocorrelation curves, solid line = fitted curve. Curves normalised to 1 to facilitate comparison. **c** Left panel: EPR spectrum of bis-(2,2,5,5-tetramethyl-3-imidazoline-1-oxyl-4-yl) disulphide-labelled OmpF reconstituted in PMOXA-PDMS-PMOXA polymersomes (black line). **c** Right panel: bis-(2,2,5,5-tetramethyl-3-imidazoline-1-oxyl-4-yl) disulphide-labelled OmpF reconstituted in PMOXA-PDMS-PMOXA polymersomes and incubated with 10 mM DTT experimental (black line) and simulated (blue line). **d** Amplex Ultra Red conversion of HRP-loaded polymersomes: immediately after addition of 30 mM GSH (left), and 1 h after addition of 30 mM GSH (right). OmpF-S-S-CF equipped HRP-loaded polymersomes (green) and OmpF-SH equipped HRP-loaded polymersomes (blue). Error bars present standard deviations in activity between three separately prepared catalytic nanocompartments (*n* = 3)

The effect of different concentrations of individual components on the functionality of the final system has already been reported for the insertion of OmpF wild type into PMOXA-PDMS-PMOXA polymersomes and enzyme encapsulation within the inner cavity^53^. Here we used the optimised conditions and adapted them for the modified OmpF and our AOs. PMOXA_6_-PDMS_44_-PMOXA_6_ copolymers spontaneously self-assembled in the presence of HRP, HRP and OmpF-S-S-CF, or HRP and OmpF-S-S-NO, and hollow spherical compartments were identified by cryo-TEM (Fig. 3a, Supplementary Figure 4). These spherical polymer assemblies were demonstrated by light scattering to be polymersomes with: *R*_*H*_ of 99 ± 2 nm for HRP-loaded polymersomes containing OmpF-S-S-CF, *R*_*H*_ of 89 ± 4 nm for polymersomes loaded with HRP and equipped with OmpF-SH, and *R*_*H*_ of 101 ± 1 nm for HRP-loaded polymersomes (Supplementary Tables 1 and 2). Standard deviations were determined based on Pearson’s coefficient of the correlation function and the Guinier fitted one. The polymersome architecture was not affected by 30 mM GSH, with structural parameters *ρ* (*ρ* = *R*_*G*_/*R*_*H*_) values in the range 0.90–0.96, which confirmed a hollow sphere morphology^54^ (Supplementary Tables 1 and 2). HRP-loaded polymersomes, HRP-loaded polymersomes equipped with OmpF-SH, and HRP-loaded polymersomes equipped with OmpF-S-S-CF all preserved their size and did not aggregate after 2 weeks storage at 4 °C in the dark (Supplementary Figures 5–7).

Insertion of channel proteins into enzyme-loaded PMOXA_6_-PDMS_44_-PMOXA_6_ polymersomes is critical for in situ activity of the encapsulated enzyme, because the channels allow substrates and products of the enzymatic reaction to pass through the membrane. As OmpF is a pore protein, its functionality is independent of its orientation inside the membrane, and the channel porin mediates the flow of molecules up to 600 Da.

We evaluated OmpF-S-S-CF and OmpF-S-S-NO insertion into the polymersome membrane using FCS and EPR, respectively. A diffusion time of *τ*_d_ = 2573 ± 960 µs was obtained by FCS for polymersomes with reconstituted OmpF-S-S-CF, indicating that the modified protein gates were successfully inserted into the polymer membranes (free OmpF-S-S-CF in 1% OG has *τ*_d_ = 588 ± 261 µs). Standard deviation of the diffusion times is acquired from individual measurements (*n* = 60). By comparing the molecular brightness of the free fluorophore (CPM = 2.2 ± 0.7 kHz) and the OmpF-S-S-CF equipped polymersomes (CPM = 18.9 ± 11.1 kHz), it was calculated that there were five OmpF-S-S-CF porins/polymersome; these values are similar to those reported previously for wild-type OmpF^36^ (Fig. 3b).

HRP-loaded polymersomes containing OmpF-S-S-NO produced a broad EPR spectrum (Fig. 3c), indicative of low mobility, a result similar to that reported for 5-DSA and 16-DSA inserted in polymersomes membranes^55^. However, when these HRP-loaded polymersomes containing OmpF-S-S-NO were exposed to reductive conditions (10 mM DTT), an isotropic EPR spectrum (*a*_*N*_ = 15.9 G) was observed superimposed on the broad peak, indicating successful cleavage of some of the nitroxide spin probe from the OmpF (Fig. 3c).

### Stimuli-responsiveness of the catalytic nanocompartments

The effect of an external stimulus on the functionality of the HRP-loaded polymersomes equipped with OmpF-S-S-CF was evaluated by their response to the addition of 30 mM GSH. The fluorescent signal associated with formation of a resorufin-like product (RLP) during the in situ enzymatic reaction in the presence of Amplex Ultra Red (AR) as a substrate for HRP was measured spectroscopically^56^. Enzymatic turnover of the AR substrate was significantly lower with HRP-loaded polymersomes equipped with OmpF-S-S-CF (by up to 36±4%) compared to HRP-loaded polymersomes equipped with OmpF-SH, suggesting that the molecular cap is sufficient to reduce the passage of small molecules through the pore. Note that the very low activity of HRP-loaded polymersomes without inserted OmpF was taken into account for background correction. Standard deviation is based on three measurements of separately prepared catalytic nanocompartments. Addition of 30 mM GSH to the system increased the activity of HRP-loaded polymersomes equipped with OmpF-S-S-CF up to that of HRP-loaded polymersomes equipped with OmpF-SH. This indicates that reduction of the disulphide bridge between the attached SAMSA-CF cap and cysteine residues of the OmpF-M successfully restored the OmpF-M pore permeability for the substrate of the enzyme by releasing the molecular cap (Fig. 3d, Supplementary Figures 8 and  9).

### Nanocompartments as stimuli-responsive AOs

Here we have gone a step further by developing stimulus-triggered AOs, whose functionality is modulated by the responsiveness of modified OmpF porins inserted in the membrane of the catalytic nanocompartments. Previously designed AOs successfully overcame the first barrier of cell membranes and escaped from endosomes^17^. As PMOXA-PDMS-PMOXA polymersomes are stable at acidic pH^27,36^, we consider that this will favour a successful lysosomal/endosomal escape during the recycling of lysosomes and endosomes.

Possible internalisation mechanisms of various PMOXA-PDMS-PMOXA-based polymersomes and their high cytocompatibility in various cell lines have already been reported^17,57–59^. Here, we evaluated the cytocompatibility of the biomimetic AOs by testing their cellular toxicity using the 3-(4,5-dimethylthiazol-2-yl)-5-(3-carboxymethoxyphenyl)-2-(4-sulphophenyl)-2H-tetrazolium (MTS) assay before studying their intracellular activation and enzymatic activity. Notably, their biocompatibility at the cellular level was shown by the absence of any decrease in viability in HeLa cells even after 48 h (i.e. polymer concentration ranging from 0.25 to 0.75 mg ml^−1^) (Supplementary Figure 10).

**Fig. 4:**
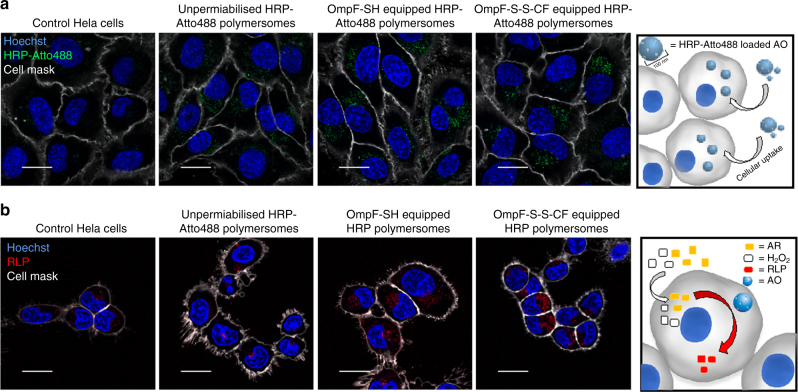
Cellular uptake and intracellular activation of AOs. **a** Confocal fluorescence micrographs of HeLa cells showing cellular uptake of fluorescently labelled HRP-loaded polymersomes and AOs loaded with fluorescently labelled HRP. Scale bar: 10 µm. **b** Cellular uptake and intracellular activation of fluorescently labelled HRP-loaded polymersomes and fluorescently labelled HRP-loaded AOs. Blue signal: Hoechst 33342 nucleus stain. Grey signal: CellMask Deep Red Plasma membrane stain. Green signal: Atto488 HRP. Red signal: resorufin-like product (RLP). Scale bar 20 µm

In order to study cellular internalisation and intracellular localisation, we first conjugated HRP with Atto488 (HRP-Atto488) and Atto647 (HRP-Atto647), respectively (Supplementary Figure 11). Then we encapsulated labelled-HRP inside the cavity of polymersomes, polymersomes equipped with OmpF-S-S-CF, and polymersomes equipped with OmpF-SH. Cellular uptake assays in HeLa cells indicated successful internalisation resulting in a particulate intracellular staining pattern with increasing intensity in a time-dependent manner from 8 to 24 h (Fig. 4a, Supplementary Figures 12 and 13). The quantitative analysis indicates that after 24 h AOs did not co-localise with early endosomes or lysosomes, confirming successful intracellular endosomal escape (Supplementary Figure 14)^17^. Localised HRP-Atto488 signals confirmed the intracellular integrity of the polymersomes. In sharp contrast, if cells were treated with a membrane disrupting agent (i.e. 0.1% saponin) (Supplementary Figure 15), polymersome membranes were affected and resulted in an intracellular cytoplasmic distribution of HRP-Atto488.

The capacity of the AOs to act within target cells in a stimuli-responsive manner was investigated by using a combination of confocal laser scanning microscopy (CLSM) and flow cytometry to evaluate their potential to respond to increased intracellular GSH levels. HeLa cells were incubated with HRP-loaded polymersomes without OmpF or with HRP-loaded polymersomes equipped with either OmpF-S-S-CF (AOs) or with OmpF-SH. Extracellular polymersomes were removed by washing before imaging the intracellular activity of AOs. Cells were incubated with a 1:1 substrate mixture of H_2_O_2_ and AR to allow the intracellular deposition, and finally conversion of AR into its RLP by AOs. Note that both hydrogen peroxide and AR pass through the cellular membrane via passive partitioning, while they do not penetrate the membrane of polymersomes (Supplementary Figure 9). In contrast to untreated cells, or those incubated with HRP-loaded polymersomes without OmpF, a significant increase of intracellular fluorescence was observed with AOs equipped with OmpF-S-S-CF or OmpF-SH (Fig. 4b, Supplementary Figure 16). A similar trend was observed when AR turnover was quantified by flow cytometry (Supplementary Figure 17). The strong fluorescent signal for AOs based on HRP-loaded polymersomes equipped with OmpF-S-S-CF confirmed successful intracellular cleavage of the molecular cap attached to OmpF-M, and subsequent activation of the AOs within the intracellular environment of the HeLa cells (Supplementary Figure 17).

### In vivo activity of stimuli-responsive AOs

**Fig. 5:**
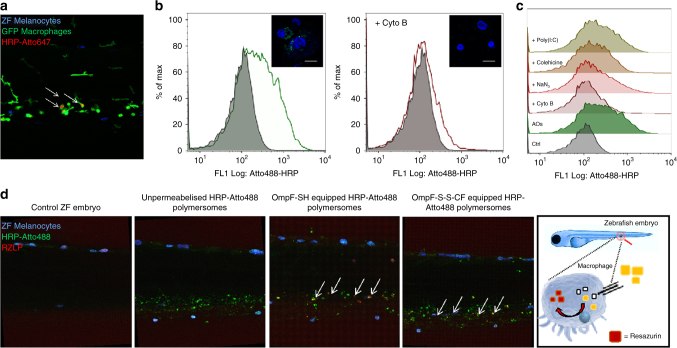
Internalisation and activity of AOs in macrophages in vitro and in vivo. **a** Localisation of AOs in ZFE. Lateral view of the ZFE injected with HRP-Atto647-loaded AOs equipped with OmpF-S-S-CF. Arrowheads: Localisation of AOs. Blue signal: ZFE melanocytes. Green signal: GFP macrophages. Red signal: Atto647-loaded AOs. **b** Phagocytosis of AOs by human macrophage differentiated THP-1 cells in vitro. Qualitative (inset) and quantitative analysis of macrophage differentiated THP-1 cells incubated with AOs loaded with Atto488-labelled HRP without addition of inhibitors (left), and in the presence of phagocytosis inhibitor cytochalasin B (Cyto B) (right). Blue signal: Hoechst 33342 nucleus stain. Green signal: Atto488 HRP. Scale bar inset 20 µm. **c** Quantification of AOs in the presence of different pharmacological pathway inhibitors by flow cytometry: polyinosinic acid (Poly(I:C)), colchicines, cytochalasin B (Cyto B) and sodium azide (NaN_3_). **d** In vivo ZFE biodistribution and activity of AOs—lateral view of the ZFE cross-section. Blue signal: ZFE melanocytes. Green signal: HRP-Atto488. Red signal: Resazurin-like product (RZLP). Arrows show regions of enzymatic activity of AOs

As a step further to obtaining insight into their safety, tolerability and performance in vivo, AOs were studied in a ZFE model. ZFEs were selected, because of their recognition as a complementary vertebrate animal model for applications, such as compound screening in drug discovery, toxicological studies and recombinant disease models^60–62^. Compared to rodent in vivo models, the ZFE offers unique advantages: (i) high reproducibility, (ii) low costs, (iii) high level of genetic homology to humans, (iv) availability of transgenic lines and (v) most importantly for the evaluation of AO, optical transparency. Due to their optical transparency, ZFE provide the possibility of imaging fluorescently-tagged objects and fluorescent processes in vivo at a high resolution over time^63^ (Supplementary Figure 18). Our approach offers the possibility of gaining detailed insight into the circulation behaviour of AOs and subsequent enzymatic reactions as we reported recently for nano-particulate drug delivery systems in vivo^64^. In order to follow the biodistribution of AOs, we injected intravenously via the duct of Cuvier HRP-Atto488-loaded polymersomes with membranes equipped with OmpF-S-S-CF or with OmpF-SH, respectively. No acute toxicity, such as change in behaviour i.e. mobility, seizures, heart failure or other toxic effects such as malformations, denaturation of tissue fluids or yolk mass was observed in ZFE injected with AOs after 24 h. ZFE analysed 2 h post intravenous injection of all types of AOs containing Atto488 conjugated HRP showed a distinct fluorescent staining pattern (Supplementary Figure 19) in the posterior cardinal vein region, and we hypothesise that polymersomes are recognised by the ZFE early immune system and are subsequently taken up by macrophages^65^. The remarkable recognition of polymersome-based AOs by the ZFE immune system was confirmed by the colocalisation of AOs loaded with Atto647-conjugated HRP (Atto647-HRP) injected into transgenic ZFE specifically expressing eGFP in macrophages (Fig. 5a, Supplementary Figure 20). In strong contrast to AOs loaded with Atto647-HRP, the free Atto647-HRP enzyme did not show significant macrophage colocalisation after 24 h, even when Atto647-HRP was injected at concentrations of 0.2 mg ml^−1^ (Supplementary Figure 21). Notably, only macrophages in circulation were targeted and not tissue resident macrophages (i.e. star shaped).

Once cellular internalisation of AOs by the early immune system of ZFE was successful in vivo, we explored the uptake rate, exact intracellular localisation and internalisation mechanisms of AOs in immune cells in vitro by using human macrophage differentiated THP-1 cells. AOs internalisation started as early as 30 min, and a strong internalisation by immune cells was achieved after 3 h (Supplementary Figure 22), with increasing uptake rates at higher time points. As THP-1 cells are immature macrophages with reduced phagocytotic capacity, a higher uptake rate of AOs is possible for mature (primary) macrophages in vitro and in vivo.^66^ Importantly, all macrophage uptake studies were performed in the presence of serum proteins to mimic physiological conditions in vivo because opsonisation of nanoparticles by serum proteins can highly influence their interaction with cells.^67^ To obtain a mechanistic understanding of the internalisation process, THP-1 macrophages were pre-treated with different pharmacological pathway inhibitors.^67^ We used inhibitors with specific inhibition profiles: (i) polyinosinic acid to block scavenger receptors, (ii) colchicine to inhibit pinocytosis, (iii) cytochalasin B as phagocytosis inhibitor and (iv) sodium azide to inhibit all energy-dependent uptake processes. Cells not incubated with Atto488 HRP-loaded AOs served as a control. A 1.28-fold increase in the mean fluorescence intensity (MFI) was observed by flow cytometry analysis of the cells incubated with Atto488 HRP-loaded AOs for 6 h, which indicates internalisation of AOs by THP-1 macrophages. The uptake of AOs by macrophages was significantly inhibited by cytochalasin B (a 0.13-fold increase in MFI) and in a lower degree by sodium azide (0.43-fold increase in MFI), which indicates an energy-dependent phagocytotic internalisation process (Fig. 5b, c). On the contrary, polyinosinic acid did not inhibit the AOs uptake, suggesting little or no involvement of the scavenger receptor in the internalisation mechanism of AOs (Fig. 5c).

The internalisation process analysed by CLSM using LysoTracker™ Red DND-99 as a reporter for the lysosomal compartments indicates that AOs co-localise with lysosomal compartments during their internalisation process (Supplementary Figure 23). Interestingly, we could not detect a lysosome signal (lysotracker) 24 h after incubation of macrophages with AOs, suggesting the presence of an intracellular lysosomal escape mechanism once the AOs are taken-up by macrophages (Supplementary Figure 23). After internalisation in macrophages, the signals associated with Atto488-HRP-loaded AOs in lysosomal compartments changed to larger intracellular vesicular signals. This suggests an expansion of the AO-bearing lysosomal compartments before the AOs are released into the cytosol. For an exact mechanism by which AOs escape the lysosomal compartment and interact with cellular membranes, further investigations are planned but they are beyond the scope of this study.

In order to assess in vivo stability, integrity and functionality of AOs when exposed to the conditions in the macrophage microenvironment, we performed a second injection of AOs together with the enzyme substrate AR. Injection of the co-substrate H_2_O_2_ in combination with AR was not necessary, since macrophages have the ability to produce H_2_O_2_. In addition, co-injection of H_2_O_2_ resulted in a red colouring of the whole blood volume, presumably due to haemolysis and thus interaction of AR with erythrocyte enzymes or haemoglobin^66^ (Supplementary Figure 24). Distinct colocalisation within macrophages of the converted AR oxidation product was found only for HRP-Atto488-loaded AOs equipped with either OmpF-SH or OmpF-S-S-CF: the molecular cap of OmpF-S-S-CF was cleaved in vivo leading to activation of the AOs. In sharp contrast, HRP-Atto488-loaded polymersomes without OmpF remained inactive, demonstrating that the polymersome membrane is sufficiently robust to remain intact in ZFE macrophages (Fig. 5d).

## Discussion

Design and development of AOs able to function inside cells and support the natural organelles is a necessary step towards the creation of cellular implants. Complementary as a concept to that of protocells, AOs respond to an essential need to offer efficient solutions for improved therapeutic and diagnostic options. Previously reported examples of AOs were based on confined spaces for reactions by compartmentalisation of enzymes inside nanoscale assemblies, but were not able to function in a stimuli-responsive manner. Here, we introduce a strategy to develop AOs with functionality that can be switched on by changes in the cellular microenvironment. These stimuli-responsive AOs are created by simultaneous encapsulation of an enzyme involved in the cellular redox homoeostasis and insertion of a genetically engineered channel porin to serve as a protein gate that triggers the enzymatic activity inside AOs.

Our AOs preserved their architecture and were activated after reaching the cellular microenvironment. More exciting, they are functional in a vertebrate ZFE model, which proves that the concept of AOs as cellular implants is feasible in vivo. Furthermore, stability, biocompatibility and low toxicity of AOs represent real advantages for medical applications compared to existing solutions for enzyme replacement, such as direct enzyme delivery and transfection^67^.

We believe that in the future, the high versatility of our strategy will allow straightforward development of a large variety of AOs for specific medical applications by changing the encapsulated enzymes and/or of the stimuli-responsive property of the protein gates. However, a careful selection of substrates is required to overcome the limited ability to transit through the plasma membrane of specific substrates, which are commonly used in bulk enzymatic reactions.

This example of AOs activated by changes in cellular microenvironment and that remains functional in vivo, opens the perspective of complex in situ reactions inside AOs, and represents an important advance towards the generation of multifunctional systems that will support the development of personalised medicine.

## Methods

### OmpF expression and extraction

The OmpF K89 R270 cysteine mutant and the OmpF wild type were expressed in BL21 (DE3) Omp8 *Escherichia coli* cells; detailed procedure is described in Supplementary Methods.^42^ The extracted fraction was analysed by a 4–15% Mini-PROTEAN^®^ TGX™ Precast SDS (Bio-Rad Laboratories, USA) gel to confirm the protein purity and the protein concentration was determined using a BCA assay kit (Pierce Chemical Co, Rockford, USA). OmpF was stored at 4 °C in 3% OG at a concentration of 1.2 mg ml^−1^ for several weeks.

### OmpF modification with SAMSA fluorescein (OmpF-S-S-CF)

The OmpF K89 R270 double cysteine mutant was modified by disulphide binding of SAMSA fluorescein to the free cysteine residues. The same reaction was also performed in the presence of OmpF wild type in 3% octyl-glucopyranoside (OG) (Anatrace, USA) and 3% OG in order to serve as controls. Twenty microlitres of 959 µM SAMSA-CF (5-((2-(and-3)-*S*-(acetylmercapto) succinoyl) amino) fluorescein) (Thermo Fischer Scientific) dissolved in 5% DMSO, 1% OG in PBS buffer was added to 400 µl of 0.4 mg ml^−1^ OmpF. The mixture was shaken in dark conditions for 30 min, when deprotection of SAMSA-CF was initiated by adjusting the pH of the solution to 8.5 with 0.5 M NaOH. The reaction mixture was incubated and shaken in the absence of light for 24 h at room temperature, after which another 5 µl of 959 µM SAMA fluorescein was added. Twenty-four hours after the second addition of SAMSA fluorescein the protein was purified from the reaction mixture by washing 25 times with 1% OG in PBS pH 7.4 in Amicon Ultra-0.5 ml centrifugal filters for protein purification and concentration, molecular cut-off: 30 kDA (Millipore) (10 min at 13,000 RPM). The volume was adjusted to 475 µl with PBS pH 7.4, and the protein concentration was determined by UV-Vis spectroscopy. Forty microlitres of the purified protein fraction was taken for FCS analysis and SDS gel electrophoresis. The volume was adjusted to 500 µl and the protein was dialysed against 1 l of 0.05 % OG in PBS for 16 h and twice against PBS for 2 h using 14 kDa Membra-Cel^TM^ (Carl Roth, Germany) dialysis membranes. The protein concentration was verified by UV-VIS (A280) (Thermo Fischer Scientific, Switzerland).

### OmpF modification with (bis-(2,2,5,5-tetramethyl-3-imidazoline-1-oxyl-4-yl) disulphide) (OmpF-S-S-NO^*^)

The OmpF K89 R270 double cysteine mutant was modified by disulphide binding of (bis-(2,2,5,5-tetramethyl-3-imidazoline-1-oxyl-4-yl) disulphide) (Noxygen, Germany) to the free cysteine residues. The same reaction was also done in presence of OmpF wild type in 3% OG PBS and 3% OG PBS in order to serve as controls for unspecific binding of (bis-(2,2,5,5-tetramethyl-3-imidazoline-1-oxyl-4-yl) disulphide) to wild-type OmpF and unspecific interactions with OG micelles. Twenty microlitres of dissolved (bis-(2,2,5,5-tetramethyl-3-imidazoline-1-oxyl-4-yl) disulphide) (1.4 mM) in 4% DMSO, 1% OG in PBS buffer were added to 400 µl of 0.4 mg ml^−1^ OmpF and mixed. The reaction was performed as described above for OmpF-S-S-CF. Twenty-four hours after the second addition of (bis-(2,2,5,5-tetramethyl-3-imidazoline-1-oxyl-4-yl) disulphide) the conjugated protein was purified from the reaction mixture by washing it 25 times with 1% OG in PBS at pH 7.4 using Amicon Ultra-0.5 ml centrifugal filters for protein purification and concentration; molecular cut-off: 30kDA (Millipore). The volume was adjusted to 475 µl using PBS at pH 7.4, and the protein concentration was determined by UV-Vis spectroscopy. Forty microlitres of the purified protein fraction was taken for EPR analysis. The volume was adjusted to 500 µl and the protein dialysed against 1 l of 0.05 % OG in PBS for 16 h and twice against PBS for 2 h using 14 kDa Membra-Cel^TM^ (Carl Roth, Germany) dialysis membranes. The protein concentration was verified by UV–VIS (A280) (Thermo Fischer Scientific, Switzerland).

### Characterisation of SAMSA fluorescein conjugated OmpF

A 4–15% Mini-PROTEAN^®^ TGX™ Precast SDS (Bio-Rad Laboratories, USA) gel polyacrylamide gel was used, then samples were mixed with BN-PAGE loading buffer and 15 µl of the final OmpF solution was added to the gel. To show the effect of GSH, separate probes were incubated with the loading buffer supplemented with 30 mM GSH. The gels were run at 200 V for 45 min. (Supplementary Figure 2), and scanned unstained and stained with Coomasie blue.

### Fluorescence correlation spectroscopy

All FCS measurements were performed using a Zeiss LSM 510-META/Confocor2 (Carl Zeiss, Jena, Germany) with an argon laser (488 nm), and ×40 water immersion C-Apochromat Objective lens. Measurements were performed at room temperature using a sample volume of 20 µl on a covered Lab-Tek Nunc^®^ Lab-Tek^®^ II chambered cover glass (Nalage Nunc International). Measurements were recorded over 3 s, and each measurement was repeated 60 times. The structural parameter and the diffusion time of the free dye in PBS pH 7.4 (SAMSA-CF) were determined independently. The autocorrelation function was calculated using a software correlator, and fitted with a one component fit (LSM 510 META-ConfoCor2 System). Detailed description of the FCS measurements and calculations are described in the Supplementary Methods.

### Preparation of reduction-triggered catalytic polymersomes

To produce reduction-triggered catalytic PMOXA-*b*-PDMS-*b*-PMOXA nanocompartments, triblock copolymer films with different subsets of Outer membrane protein F (OmpF) were rehydrated with HRP. The detailed preparation technique and control experiments are described in the Supplementary Methods.

### Characterisation of catalytic nanocompartments

The size and morphology of the stimuli-responsive catalytic nanocompartments were characterised by a combination of light scattering (SLS, DLS) and cryogenic transmission electron microscopy (Cryo-TEM). Detailed procedures are described in detail in the Supplementary Methods.

### Enzymatic assay

The emission fluorescence intensity was determined using a LS 55 Fluorescence Spectrometer (Perkin Elmer). Samples were incubated with a final concentration of 30 mM GSH in PBS at pH 7.4, and the pH was kept at this value. For the measurement, 10 µl of the samples mixed with GSH were transferred to 220 µl of the reaction mixture (4.5 µM H_2_O_2_ and 3.4 µM AR) in PBS at pH 7.4. The reaction mixture was excited at 530 nm and the emission intensity was monitored at 590 nm. Fluorescence was expressed as relative fluorescence units and was measured at the same instrument setting in all experiments. The detailed procedure is described in the Supplementary Methods.

### Cell toxicity assay

The [3-(4,5-dimethyl-2-yl)-5-(3-carboxymethoxyphenyl)-2-(4-sulphophenyl)-2H-tetrazolium (MTS) assay (Promega) was used to determine cell viability. HeLa cells were seeded in a triplicate at a density of 2.5 × 10^3^ cells per well in a 96-well plate. Cells were cultured for 24 h in Dulbecco’s modified Eagle’s medium (DMEM) growth medium (supplemented with 10% foetal calf serum, penicillin (100 units ml^−1^) and streptomycin (100 µg ml^−1^)). After 24 h, the medium 100 μl aliquots containing the corresponding concentration of samples [0.25, 0.5 and 0.75 mg ml^−1^] were added to the cell medium. Cells incubated only in medium served as control (100%). After 24 h of incubation 20 µl of MTS solution was added to each well. The plates were incubated for 1 h at 37 °C, and absorption was measured at *λ* = 490 nm. The quantity of formazan product as measured by absorbance at 490 nm is directly proportional to the number of living cells in the culture. Absorption of cells where no nanoparticles were added served as 100%.

### 24 h uptake of catalytic nanocompartments

HeLa (epitheloid cervix carcinoma, human; ATCC, CCL-2) cells were cultured at a density of 3 × 10^4^ cells per well in an eight-well Lab-Tek (NalgeNunc International, USA) for 24 h in DMEM growth medium supplemented with 10% foetal calf serum, penicillin (100 units ml^−1^) and streptomycin (100 µg ml^−1^) to allow attachment to the surface. After attachment, the medium was removed and catalytic nanocompartments were added to a final polymer concentration of 0.5 mg ml^−1^. Cells were washed twice before being imaged at the respective time points.

### Flow cytometry analysis of AO activity

HeLa (epitheloid cervix carcinoma, human; ATCC, CCL-2) cells were seeded in a well of a 24-well plate (8 × 10^4^ cells per well) and cultured in DMEM containing 10% foetal calf serum, penicillin (100 units ml^−1^) and streptomycin (100 µg ml^−1^)) for 24 h at 37 °C in a humidified CO_2_ incubator. Then the medium was exchanged and polymersome solution was added to a final concentration of 0.5 mg ml^−1^ for another 24 h. Cells were washed three times with PBS, trypsinised, centrifuged, washed, centrifuged and then suspended in 1 ml PBS. AR/H_2_O_2_ was added to a final concentration of 10 µM, and after 2 h, flow cytometry analysis was performed using a BD FACSCanto II flow cytometer (BD Bioscience, USA). Doublets were excluded using FSC and SSC detectors, single cells were excited at 561 nm and the emission was detected in FL5 (586/15; Resorufin Channel). A total of 10,000 single cells for each sample were analysed, and data processed using Flow Jo VX software (TreeStar, Ashland, OR).

### Intracellular stability of AO

HeLa (epitheloid cervix carcinoma, human; ATCC, CCL-2) cells were seeded at a density of 3 × 10^4^ cells ml^−1^ onto poly-d-lysine-coated glass coverslips. Cells were cultured for 24 h in DMEM growth medium (supplemented with 10% foetal calf serum, penicillin (100 units ml^−1^) and streptomycin (100 µg ml^−1^)). After attachment to the surface, the medium was removed and catalytic nanocompartments were added to a final polymer concentration of 0.5 mg ml^−1^. Cells were incubated for an additional 24 h in the medium, then washed three times with PBS and fixed with 4% PFA for 15 min. After a neutralisation step using 50 mM NH_4_Cl, cells were either treated with PBS (control) or 0.1% saponin for 10 min at room temperature. After additional washing steps, cell nuclei were counterstained for 10 min using Hoechst 33342 (0.5 µg ml^−1^). Finally cells were embedded in Vectashield antifade mounting media. CLSM was performed using an Olympus FV‑1000 inverted microscope (Olympus Ltd, Tokyo, Japan) equipped with a ×60 UPlanFL N oil‑immersion objective (numerical aperture 1.40). Cells were excited at 405 nm (Hoechst 33342) and 488 nm (Atto488-HRP), and the fluorescence signal was collected using Kalman modus between 425 and 475 nm and 500 and 600 nm, respectively. To minimise spectral cross talk, the samples were scanned using sequential mode. The laser settings were adjusted depending on the treatment. Images were processed using the Fiji open source image processing package of ImageJ.

### Intracellular localisation of AO

HeLa (epitheloid cervix carcinoma, human; ATCC, CCL-2) cells were seeded at a density of 3 × 10^4^ cells per well onto poly-d-lysine-coated glass coverslips. Cells were cultured for 24 h in DMEM growth medium (supplemented with 10% foetal calf serum, penicillin (100 units ml^−1^) and streptomycin (100 µg ml^−1^)) to allow attachment to the surface. After attachment, the medium was removed and catalytic nanocompartments were added to a final polymer concentration of 0.5 mg ml^−1^. After 24 h cells were washed three times with PBS and then fixed for 15 min using 4% PFA. After a neutralisation step using 50 mM NH_4_Cl, cells were incubated with either rabbit polyclonal anti-EEA1 (ab2900, Abcam) (1:1000) or anti-LAMP1 (ab24170, Abcam) antibody (1:1000) for early endosome or lysosome staining, respectively^58^. The cells were washed with PBS, followed by staining with the secondary goat anti-rabbit polyclonal Dylight633-labelled antibody (1:1000; #35562, Thermo Fisher Scientific). Cell nuclei were counterstained for 10 min using Hoechst 33342 (0.5 µg ml^−1^). Finally, cells were embedded in Vectashield antifade mounting media. CLSM was performed using an Olympus FV‑1000 inverted microscope described above. Z-stacks were taken using Kalman modus and a step size of 450 nm. Cells were excited at 405 nm (Hoechst 33342), 488 nm (Atto488-HRP), and 633 nm (Early Endosome/Lysosome). The fluorescence signal was collected between 425 and 475, 500 and 600, and 655 and 755 nm, respectively, by scanning in the sequential mode, and processed as described above.

Colocalisation of polymersomes with markers of early endosome (EEA1) or lysosome (LAMP1) were carried out using the JaCoP plug-in in the Fiji software. Pearson’s correlation coefficient (PCC), Mander’s coefficients (M1/M2, using thresholds of *A* = 200 and *B* = 180), and Costes’ randomisation-based colocalisation (200 randomisation rounds) were used to assess the extent of colocalisation.

### In cellulo activity of AOs by CLSM

HeLa cells (epitheloid cervix carcinoma, human; ATCC, CCL-2) were cultured at a density of 3 × 10^4^ cells per well in an eight-well Lab-Tek (NalgeNunc International, USA) for 24 h in DMEM growth medium (supplemented with 10% foetal calf serum, penicillin (100 units ml^−1^) and streptomycin (100 µg ml^−1^) to allow attachment to the surface. After attachment, the medium was removed and catalytic nanocompartments were added to a final polymer concentration of 0.25 mg ml^−1^. Cells were then incubated for an additional 24 h in medium, washed twice with PBS and AR/H_2_O_2_ added in the ratio of 1:1 to a final concentration of 10 µM in DMEM-based growth medium. After 30 min, cells were washed three times with PBS and their nuclei counterstained for 10 min using Hoechst 33342 (0.5 µg ml^−1^). Cells were washed twice with D-PBS and cultured in DMEM. CellMask Deep Red Plasma membrane stain (0.5 µl ml^−1^) was added and cells were analysed after 5 min. CLSM was performed as described in the previous section. The laser settings for RLP, the photomultiplier tube gain and the pinhole settings were kept constant during the analysis. Images were processed using Olympus FluoView software (v3.1, Olympus).

### In vivo activity of AOs

Standard ZFE culture medium at pH 7.4 was prepared at final concentrations of 5 mM sodium chloride, 0.25 mM potassium chloride, 0.5 mM magnesium sulphate, 0.15 mM potassium dihydrogen phosphate, 0.05 mM sodium phosphate dibasic, 0.5 mM calcium chloride, 0.71 mM sodium bicarbonate and 0.001% (w/v) methylene blue.

Collected eggs from adult ABC/TU ZFE (wild type) and EGFPs843 ZFE (GFP-macrophage line) were kept in ZFE culture medium at 28 °C. PTU (0.03 mg ml^−1^) was added 1-day post fertilisation (dpf) in order to avoid pigment cell formation. Three different enzyme-loaded polymersomes were injected into 2-dpf ZFE according to an adapted protocol originally designed for microangiography. ZFE were anaesthetised using 0.01% tricaine (w/v) and cast into 0.3% (w/v) agarose containing the same amount of tricaine. Immobilised ZFE were injected with either with 3 nl of 0.2 mg ml^−1^ free HRP or 3 nl AO solution (5 mg ml^−1^), removed from the agarose and kept in ZFE culture medium containing PTU for 24 h. Then, a second injection of 1 nl AR (78 µM) was performed following the same procedure. As control experiments, ZFE were injected with the enzymatic substrate AR and AR mixed with H_2_O_2_ without previous AO injection. Fluorescence imaging of injected ZFE was performed using an Olympus FV1000 confocal microscope (Olympus Schweiz AG, Volketswil, Switzerland). ZFE were excited at 488 nm (Atto488 HRP), 559 nm (Melanocytes) and 635 nm (Resazurin-like product) and the fluorescence signal was collected between 500 and 530, 575 and 620, and 655 and 755 nm, respectively.

### Monocyte cell culture and differentiation to macrophages

THP-1 cells (ATCC, TIB 202) were cultured at a starting density of 2 × 10^5^ cells ml^−1^ in Roswell Park Memorial Institute (RPMI-1640) medium containing 10% FCS, penicillin (100 units ml^−1^)/streptomycin (100 µg ml^−1^), 10 mM HEPES, 1% sodium pyruvate and 0.05 mM mercaptoethanol. For uptake studies, THP-1 cells were seeded at a density of 5.5 × 10^4^ cells per well onto poly-d-lysine-coated Ibidi 8-Well µ-Slides or 2 × 10^5^ cells per ml into a 12-well plate (TPP, Switzerland) for CLSM or flow cytometry, respectively. Differentiation of human monocytic cell line THP-1 to macrophages was induced 24 h after seeding using 200 nM phorbol 12-myristate 13-acetate for 72 h.

### Uptake study using pathway inhibitors

The uptake mechanism of Atto488-HRP-loaded polymersomes equipped with OmpF-S-S-CF into THP-1 macrophages was investigated using different pharmacological pathway inhibitors^67^. Cells were pre-incubated using 10 µg ml^−1^ cytochalasin B (phagocytosis) for 2 h, 0.1% sodium azide (energy-dependent uptake process) for 30 min, 100 µg ml^−1^ colchicine (pinocytosis) for 2 h and 2.5 µg ml^−1^ polyinosinic acid (scavenger receptor) for 30 min, and then treated with Atto488-HRP-loaded polymersomes.

### Qualitative uptake of AOs observed by CLSM

Macrophage differentiated THP-1 cells were incubated with Atto488-HRP-loaded polymersomes equipped with OmpF-S-S-CF at a final polymer concentration of 0.25 mg ml^−1^ for specific time points as indicated. LysoTracker Red DND-99 (Invitrogen) was added to cells 1 h before imaging at a concentration of 50 nM when indicated. Cell nuclei were counterstained using Hoechst 33342 (2.5 µg ml^−1^). Cell membranes were stained using CellMask Deep Red Plasma membrane stain (0.5 µl ml^−1^) when indicated directly before imaging. Live cell imaging was performed as described in the previous section using an Olympus FV‑1000 inverted microscope (Olympus Ltd, Tokyo, Japan) equipped with a ×60 UPlanFL N oil‑immersion objective (numerical aperture 1.40). Orange colour indicated colocalisation of polymersomes with lysosomes (LysoTracker Red DND-99).

### Quantitative uptake studies by flow cytometry

Differentiated THP-1 cells were incubated with Atto488-HRP-loaded polymersomes equipped with OmpF-S-S-CF at a final polymer concentration of 0.25 mg ml^−1^ for specific time points as indicated, or in the presence of different pharmacological pathway inhibitors for 6 h. Flow cytometry analysis was performed using a BD FACSCanto II flow cytometer (BD Bioscience, USA) as described in the previous section.

### Ethical regulations

All procedures on live zebrafish embryos (Danio rerio) were carried out following the Swiss legislation on animal welfare.

### Data availability

The data that support the findings of this study are included in the Supplementary Information; the remaining data are available from the corresponding author upon reasonable request.

## Electronic supplementary material

Supplementary Information(PDF 2361 kb)
