# Supplementary material for: Biomimetic artificial organelles with in vitro and in vivo activity triggered by reduction in microenvironment
Source: Nat Commun. 2018 Mar 19;9:1127. doi: 10.1038/s41467-018-03560-x (PMC5859287; doi:10.1038/s41467-018-03560-x)
Supplement: Supplementary file 1 — Supplementary Information(PDF 2361 kb) [file 41467_2018_3560_MOESM1_ESM.pdf]

Supplementary Information

Biomimetic artificial organelles with *in vitro* and *in vivo* activity triggered by reduction microenvironment

T. Einfalt[1,2], D. Witzigmann[2], C. Edlinger[1], S. Sieber[2], R. Goers[1,3], A. Najer[1], M. Spulber[1], O. Onaca-Fischer[1], J. Huwyler[2], and C. G. Palivan[1*]

[1]Department of Chemistry, University of Basel, Klingelbergstrasse 80 CH-4056 Basel, Switzerland

[2]Department of Pharmaceutical Sciences, Division of Pharmaceutical Technology, University of Basel, Klingelbergstrasse 50 CH-4056 Basel, Switzerland

[3]Department of Biosystems Science and Engineering, ETH Zürich, Mattenstrasse 26, CH-4058 Basel, Switzerland

Corresponding Author

*E-mail: cornelia.palivan@unibas.ch

Materials

Reagents were purchased from Sigma-Aldrich unless otherwise specified.

Supplementary Methods

OmpF expression and extraction

Under aseptic conditions, cells from an ampicillin resistant *Escherichia Coli* strain BL21 stock culture overexpressing the cysteine OmpF K89 R270 mutant were smeared onto the surface of LB, ampicillin agar plate[1]. After 16h incubation at 37 °C, a single bacterial colony was transferred from the plate to 15 mL of TB liquid medium with 100 mg mL^{-1} ampicillin (total 5 flasks). 5 times 1 L of TB medium with 100 mg mL^{-1} ampicillin was inoculated with 15 mL of the overnight culture. The optical density (OD) of the growing culture was followed by measuring the absorbance at 600 nm. Once the absorbance reached an OD of 0.6, IPTG was added to a final concentration of 1 mM into each flask in order to start the over-expression of the OmpF K89 R270 gene. Bacteria were grown for 16 more hours at 25 ºC with vigorous shaking. Then the *E. Coli* cells were pelleted at 7.000 x *g* for 8 min at 4 ºC. Pellets were stored at -25 ºC, and on the day following the harvesting re-suspended in 50 mL of 25 mM Tris-HCl at pH 7.4. 10 mg DNase and 10 mg RNase were added, and the cell suspension was French pressed five times at 1,000 bar. 1 mL of 20% SDS was added per 10 mL of cell lysate and incubated for 1 h at 60 ºC. The suspension was centrifuged at 50.000 x *g* at RT. The supernatant was removed and the cell pellet incubated in 0.125% OG for 1 h at 37ºC. The suspension was centrifuged at 50.000 x *g* at RT. Finally, the cell pellet was re-suspended in 3% n-octyl-β-D-glucopyranoside (OG) (Anatrace, USA) in 10 mM phosphate buffer and homogenised. The suspension

was centrifuged at 50.000 x *g* and the protein concentration in the supernatant was determined by UV-Visible absorption spectroscopy at 280 nm.

Preparation of OmpF equipped polymersomes

OmpF equipped polymersomes were prepared at RT from the ABA triblock copolymer, $PMOXA_6$-$PDMS_{44}$-$PMOXA_6$, ($A_6B_{44}A_6$) and a subset of modified or unmodified OmpF (OmpF-SH, OmpF-S-S-$NO^{\bullet}$ and OmpF-S-S-CF). The synthetic procedure and the polymer characterization are presented in [2]. Polymersomes were generated using the film rehydration technique where the polymer was dried in the presence of native or modified OmpF K89 R270 cysteine mutant (OmpF-SH, OmpF-S-S-CF or OmpF-S-S-$NO^{\bullet}$). Horseradish peroxidase (HRP), Atto-488 conjugated HRP (Atto488-HRP) or Atto-647 conjugated HRP (Atto647-HRP) in PBS buffer pH=7.4 at RT were used as rehydration solutions. Films were rehydrated to a final polymer concentration of 2.5 mg mL^{-1}, HRP concentration of 0.2 mg mL^{-1}, and OmpF concentration of 80 µg mL^{-1}, respectively. Control polymersomes were also prepared in the absence of OmpF. Rehydrated films were stirred in the dark overnight at RT. All samples were extruded through an Avanti mini-extruder (Avanti Polar Lipids, USA) using a 100 nm diameter pore-size polycarbonate membrane (11 times) at RT in order to obtain size homogeneity. Non-encapsulated enzyme was removed from the polymersomes by dialysis against PBS at pH 7.4 at RT for 5 daysusing Spectrapore dialysis tubes, MWCO 300 kDa (Spectrum Laboratories Inc., USA); the buffer was exchanged every 3 hours during the day, but unchanged overnight. Steps involving the generation of catalytic nanocompartments were performed in the dark in order to

avoid photo degradation of fluorophores. Incubation experiments and activity measurements were performed on the day following the last dialysis step.

LC-MS-MS

Mass spectrometry was used to determine the degree of labelling of the OmpF conjugates[1]. The spectrometer consisted of a LC-MS column ReproSil-Pur C18–AQ, 1.9 µm resin Dr. Maisch GmbH, Ammerbuch-Entringen, Germany combined with a dual pressure LTQ-Orbitrap Elite mass spectrometer connected to an electrospray ion source (Thermo Fisher Scientific). The in gel digestion was based on a previously published protocol[3]. Briefly, the band of interest was cut out of the SDS-gel and the sample was cut to tiny cubes. The Coomassie Blue stain was rinsed out and the protein was alkylated with iodoacetamide and digested using trypsin. The peptides were then washed out and desalted with C18 reversed phase spin columns (Microspin, Harvard Apparatus). After drying, the samples were dissolved and subjected to LC–MS analysis using a linear gradient from 95% solvent A (0.15% formic acid, 2% acetonitrile) and 5% solvent B (98% acetonitrile, 0.15% formic acid) to 28% solvent B over 40 min at a flow rate of 0.2 µl/min. The 20 most intense ions were released from the linear ion trap and subjected to a MS-MS analysis. The intensity of the mutation carrying fragment was put in relation to another fragment of the same sample to eliminate the dependence on the concentration and the ratio was then compared to the same ratio of OmpF with free thiol groups (Supplementary Equation 1).

Supplementary Equation 1: $\left(\frac{I_{SH-fragment}}{I_{other-fragment}}\right)_{conjugate} \Big/ \left(\frac{I_{SH-fragment}}{I_{other-fragment}}\right)_{mutant}$

For three separately measured samples a labeling degree of 96±-4% (100%, 95%, 100%) degree of labelling of OmpF was determined for $NO^{\bullet}$ labelling and 81+/-31% (100%, 100%, 46%) for labelling with SAMSA-CF. Standard deviations are based on 3 measurements.

EPR measurements of OmpF-S-S-NO$^{\bullet}$

EPR measurements were performed on a Bruker CW EPR Elexsys-500 spectrometer equipped with a variable temperature unit. The spectra were recorded at 298 K with the following parameters: 100 KHz magnetic field modulation, microwave power 2 mW, conversion time 61.12 ms, number of scans up to 200, resolution 2048 points, modulation amplitude 0.5 G for the samples containing the free nitroxide and 1 G for the samples containing spin labeled OmpF (OmpF-S-S-$NO^{\cdot}$), sweep width 150 G. Note: the EPR spectra presented in the figures (Figure 1 and Figure 2) were zoomed to a maximum 80G sweep width for better resolution of the peaks. Isotropic EPR spectra were simulated using the WINSIM (NIEHS/NIH) simulation package, while the anisotropic spectra were simulated with Bruker WinEpr SimFonia package.

For the free nitroxide spin probe (bis-(2,2,5,5-tetramethyl- 3-imidazoline-1-oxyl-4-yl) disulphide), a_N values correspond to half the distance (in Gauss) between the low-field and high-field lines of the isotropic spectra (triplets).

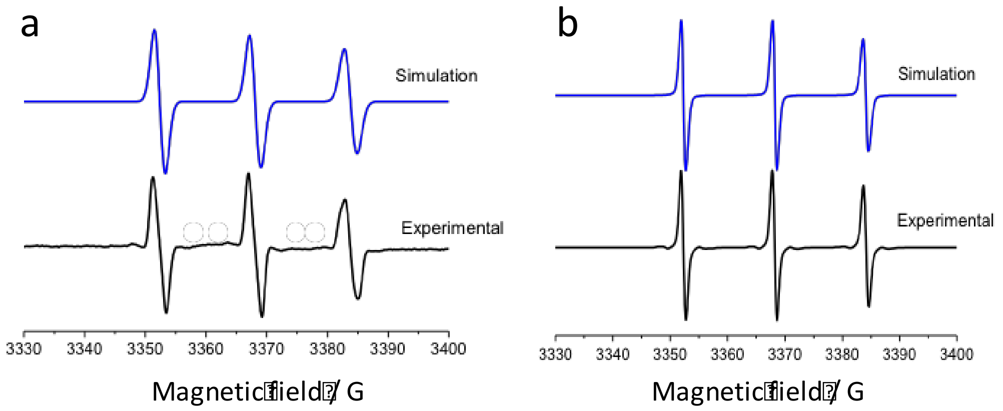

Supplementary Figure 1. EPR measurement of free bis-(2,2,5,5-tetramethyl- 3-imidazoline-1-oxyl-4-yl) disulphide (A) EPR signal of bis-(2,2,5,5-tetramethyl- 3-imidazoline-1-oxyl-4-yl) disulphide in PBS, experimental (black) and simulated (blue) (B) EPR signal of bis-(2,2,5,5-tetramethyl- 3-imidazoline-1-oxyl-4-yl) disulphide in presence of 10mM DTT experimental (black) and simulated (blue).

SDS Gel chromatography

A 4-15% Mini-PROTEAN® TGX™ Precast Gels SDS was used. OmpF-WT / OmpF K89 R270 - exposed to the activated SAMA fluorescein and OmpF-WT / OmpF K89 R270 mutant – without further modification, were mixed with Laemmni loading buffer, and 15µL of the final OmpF solutions were added to the gel. In parallel the same probes were incubated with 10mM GSH and added to cells. Gels were run at 200V for 40 minutes (Supplementary Figure 2).

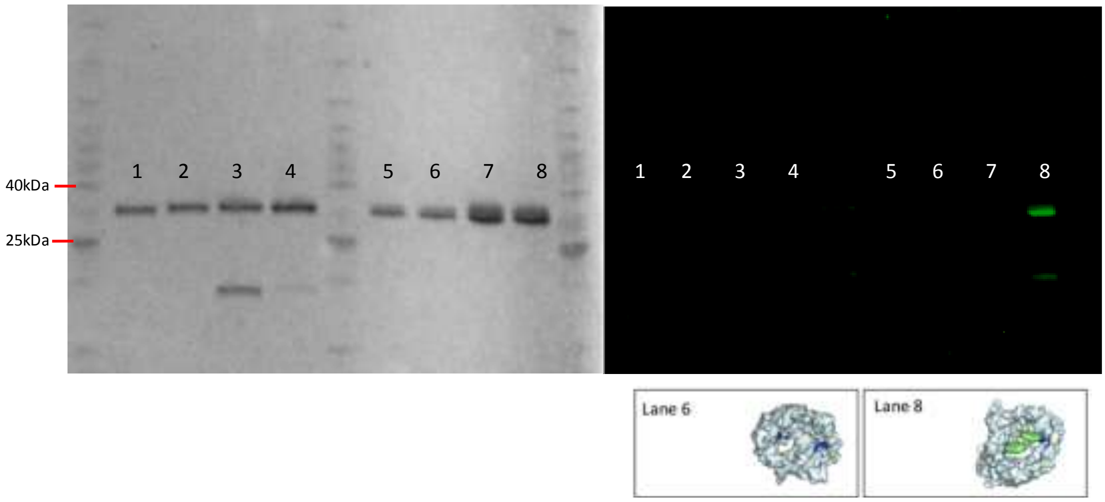

Supplementary Figure 2. **4-15% SDS-PAGE of modified and unmodified OmpF**. Left: Coomassie blue stained gel. Right: Fluorescent image. Lane 1. OmpF wild type exposed to 30mM GSH, Lane 2. OmpF wild type exposed to SAMSA- CF and 30mM GSH, Lane 3. OmpF K89 R270 exposed to 30mM GSH, Lane 4. OmpF K89 R270 exposed to SAMSA-fluorescein and 30mM GSH Lane 5. OmpF wild type, Lane 6. OmpF wild type exposed to SAMSA-fluorescein, Lane 7. OmpF K89 R270, Lane 8. OmpF K89 R270 exposed to SAMSA-fluorescein.

Fluorescence correlation spectroscopy

All FCS measurements were carried out using a ConfoCor2 instrument (Carl Zeiss, Germany) with a 40x, 1.2 water immersion C-Apochromat objective lens. Measurements were carried out at room temperature using a sample volume of 20 µL on a covered eight-well Lab-Tek chambered borosilicate cover glass (Nalage Nunc International, USA). An Argon 488 laser was used for excitation of the SAMSA-CF fluorophore at 488 nm (Laser power output 20%, 8mW) with the appropriate filter sets. The fluorescence signal was measured in real time and the autocorrelation function was calculated by a software correlator (LSM 510 META-ConfoCor 2

System). Measurements were recorded over 3s and each measurement was repeated 60 times. Correlation curves that could not be fitted were excluded (<10%).

For determining the binding of SAMSA-CF to OmpF-SH and the successful reconstitution of OmpF-S-S-CF into HRP loaded PMOXA-PDMS-PMOXA polymersomes, experimental auto correlation curves were fitted using a one component model including the triplet state (Supplementary Equation 2):

$$G(\tau)_{fit} = 1 + (1 + \frac{T}{1-T} e^{-\frac{\tau}{\tau_{trip}}}) \frac{1}{N} \left[\frac{1}{1 + \frac{\tau}{\tau_D}} \frac{1}{\sqrt{1 + R^2 \frac{\tau}{\tau_D}}} \right]$$

τ_D represents the diffusion time, T the fraction of fluorophores in the triplet state with triplet time τ_{trip}, N is the number of particles and R the structural parameter. R and τ_D of free dye SAMSA-CF were determined independently. The number of SAMSA-CF molecules per OmpF was determined by comparing the molecular brightness (CPM) of SAMSA-CF (2.2 ± 0.7 kHz) with that of SAMSA-CF covalently attached to OmpF K89 R270 (4.8 ± 0.6 kHz). Similarly, the number of OmpF-S-S-CF monomers per vesicle was determined by comparing the molecular brightness of SAMSA-CF (2.2 ± 0.7 kHz) to the molecular brightness of OmpF-S-S-CF in 1% OG (18.9 ± 11.1 kHz), taking into consideration that every OmpF monomer is modified by two SAMSA-CF molecules. Standard deviations of molecular brightness are based on individual FCS measurements (n=60).

For determining the release kinetics of SAMSA-fluorescein from OmpF-S-S-CF, experimental auto correlation curves were fitted using a two-component model including a triplet state (Supplementary Equation 3):

$$G(\tau)_{fit} = 1 + \left(1 + \frac{T}{1-T} e^{-\frac{\tau}{\tau_{trip}}}\right)\frac{1}{N}\left[\frac{f_1}{1+\frac{\tau}{\tau_{D1}}}\frac{1}{\sqrt{1+R^2\frac{\tau}{\tau_{D1}}}}\right] + \left(1 + \frac{T}{1-T} e^{-\frac{\tau}{\tau_{trip}}}\right)\frac{1}{N}\left[\frac{f_2}{1+\frac{\tau}{\tau_{D2}}}\frac{1}{\sqrt{1+R^2\frac{\tau}{\tau_{D2}}}}\right]$$

τ_D represents the diffusion time, T the fraction of fluorophores in the triplet state with triplet time τ_{trip}, N is the number of particles and R the structural parameter.

R and τ_D of free dye (SAMSA-CF) were determined independently in 30mM GSH, PBS pH 7.4, and subsequently fixed in the fitting procedure in order to determine the % of free dye, which represented the SAMSA-CF released from OmpF-S-S-CF in 1% OG 30mM GSH, PBS pH 7.4 (Supplementary Figure 3).

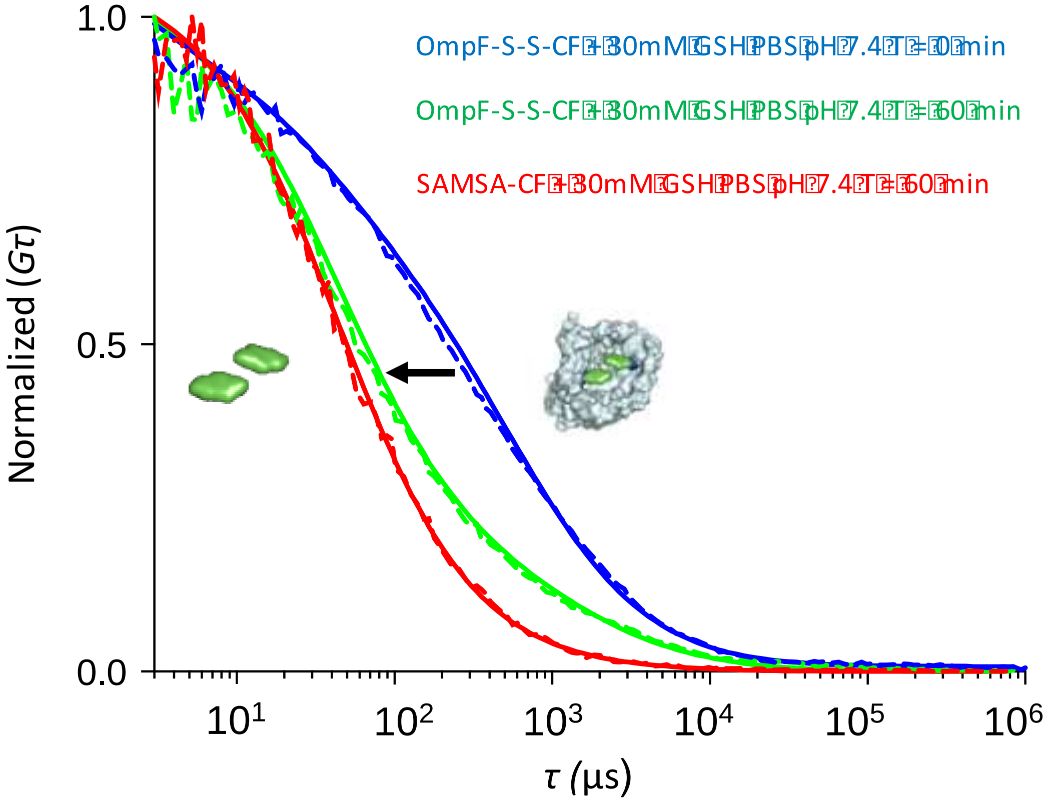

Supplementary Figure 3. FCS analysis of OmpF-S-S-CF stimuli responsiveness. FCS autocorrelation curves of SAMSA-CF in 1% OG 30mM GSH (red), OmpF-S-S-CF mixed with 30mM GSH in 1% OG Time 1h (green) and OmpF-S-S-CF in 1% OG Time 0h, PBS (blue). Dotted line – experimental auto correlation curves, Full line – fit. Curves normalised to 1 to facilitate comparison.

Cryogenic-TEM

Polymersome suspensions in buffer (10 mM PBS, pH 7.4, 50 mM NaCl) at high concentrations (2.5 mg mL^{-1}) were deposited on glow-discharged carbon grids (Quantifoil, Germany) and blotted before quick-freezing in liquid ethane using a Vitribot plunge-freezing device (FEI Co.). The grids were stored in liquid nitrogen before transferring them into a cryo-holder (Gatan). Imaging was performed on a

Philips CM200 FEG TEM at 200 kV with accelerating voltage in low-dose mode and a defocus value of about 4 µm.

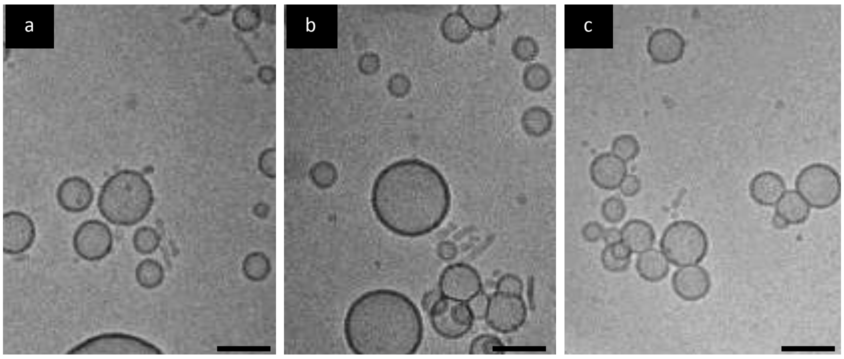

Supplementary Figure 4. Cryo-TEM overview micrographs of: (a) Polymersomes loaded with HRP and equipped with OmpF-S-S-CF, (b) polymersomes loaded with HRP and equipped OmpF-SH, and (c) polymersomes loaded with HRP. Scale bar = 100 nm

Light scattering

Dynamic and static light scattering (DLS and SLS) experiments were performed at 20 °C using an ALV/CGS–8F goniometer (Langen/Hessen, Germany) equipped with a frequency-doubled He-Ne laser (JDS Uniphase, λ = 632.8 nm). Reduction-responsive polymer nanocompartments were serially diluted to polymer concentrations ranging from 0.05 mg mL^{-1} down to 0.025 mg mL^{-1}, and measured at scattering angles between 30° and 150°, with an angular step of 10°, in 10 mm diameter cylindrical quartz cells mounted in a thermostatted optical matching toluene bath. The photon intensity auto-correlation function, $g^2(t)$, was determined with an ALV/LSE–5004 digital correlator. A nonlinear decay-time analysis supported by regularised inverse Laplace transform of $g^2(t)$ (CONTIN algorithm) was used to analyse the DLS data. The angle-dependent apparent diffusion coefficient was extrapolated to zero momentum transfer (q^2) using the ALV/Static and dynamic FIT and PLOT 4.31

software. Angle and concentration-dependent SLS data are presented as Guinier plots (Supplementary Figure 7-8) [4].

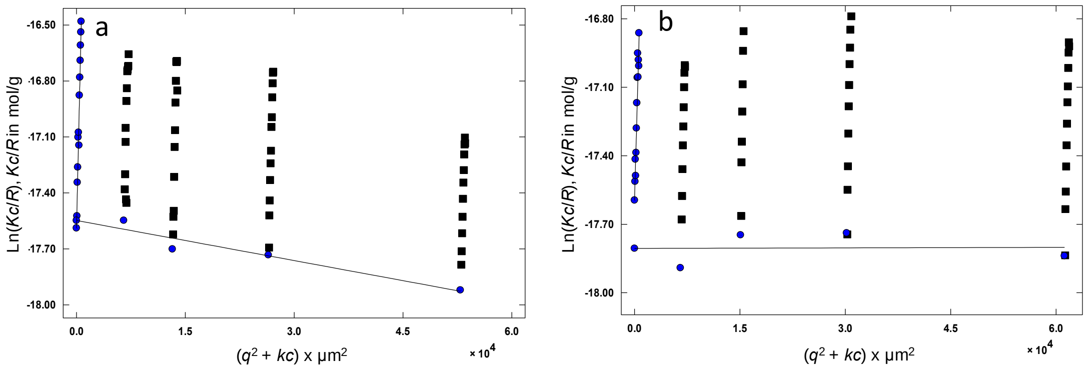

Supplementary Figure 5. **Guinier plot representation of SLS data.** HRP-loaded polymersomes equipped with OmpF-S-S-CF in PBS at pH 7.4 (a), and in the presence of 30 mM GSH in PBS at pH 7.4 (b).

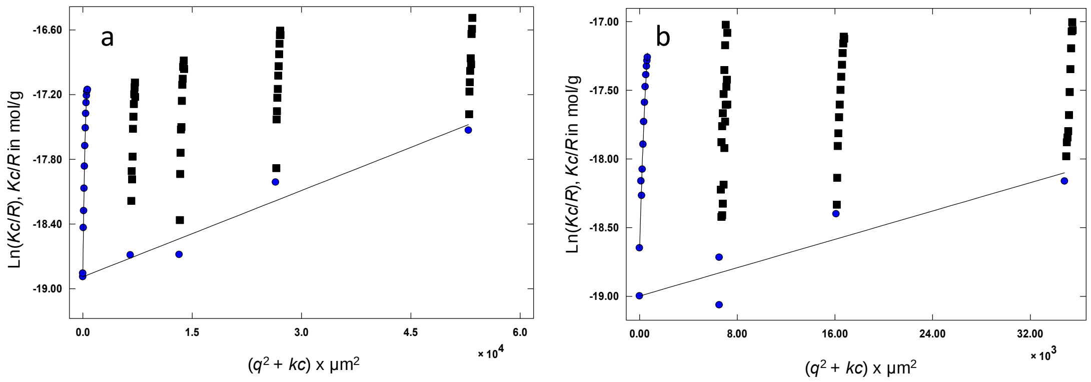

Supplementary Figure 6. Guinier plot representation of SLS data. HRP-loaded polymersomes equipped with OmpF-SH in PBS at pH 7.4 (a), and in the presence of 30 mM GSH in PBS at pH 7.4 (b).

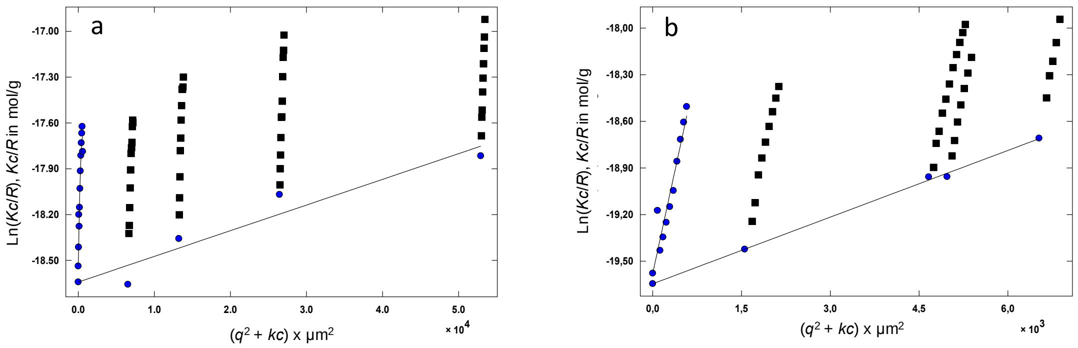

Supplementary Figure 7. Guinier plot representation of SLS data. HRP-loaded polymersomes in PBS (a), and in the presence of 30 mM GSH in PBS (b).

**Nanocompartments**	$\rho = R_g/R_h$
HRP-loaded polymersomes	0.97 ± 0.01
HRP-loaded polymersomes equipped with OmpF-S-S-CF	0.93 ± 0.02
HRP loaded polymersomes equipped with OmpF-SH	0.98 ± 0.04

Supplementary Table 1. Molecular characteristics of $PMOXA_6$–$PDMS_{44}$-$PMOXA_6$ assemblies in PBS at pH 7.4. Standard deviations were determined based on Pearson's coefficient of the correlation function and the Guinier fitted one. Mw = 4500 g/mol, PDI = 1.8, *f* (hydrophilic) = 25%

Nanocompartments	$\rho = R_g/R_h$
HRP-loaded polymersomes	0.95 ± 0.01
HRP-loaded polymersomes equipped with OmpF-S-S-CF	0.96 ± 0.02
HRP loaded polymersomes equipped with OmpF-SH	0.95 ± 0.04

Supplementary Table 2. Molecular characteristics of $PMOXA_6$–$PDMS_{44}$-$PMOXA_6$ assemblies in PBS at pH 7.4 + 30mM GSH. Standard deviations were determined based on Pearson's coefficient of the correlation function and the Guinier fitted correlation function. Mw = 4500 g/mol, PDI = 1.8, *f* (hydrophilic) = 25%

Enzymatic substrate turnover of stimuli responsive catalytic nanocompartments.

The reaction rate at which catalytic HRP-loaded polymersomes equipped with different OmpFs perform in solution was studied by employing the Amplex UltraRed enzymatic assay. Conversion of Amplex Ultra Red into its fluorescent product was determined by observing fluorescence intensity in PBS at pH 7.4 as a function of time in the presence of GSH. Linear regression was performed using Origin software, in order to identify the best linear section (Supplementary Figure 8). Substrate turnover of OmpF-S-S-CF equipped HRP-loaded polymersomes, OmpF-SH equipped HRP-loaded polymersomes and unpermeabelised HRP-loaded polymersomes was averaged between three separately prepared samples. Slopes determined from the linear regression of Amplex UltraRed conversion are a direct measure of the relative reaction speed, as the difference in fluorescence over time is directly linked to the difference in concentration of the fluorescent product[5]. For % of relative activity, slopes were compared to Amplex UltraRed conversion of OmpF-SH catalytic

nanocompartments in presence and absence of GSH. The minor decrease in total HRP substrate turnover in the presence of GSH is expected as a result of noncompetitive inhibiton of the enzyme and previously reported by [6] (Supplementary Figure 9). The significant difference between the slopes of OmpF-S-S-CF equipped HRP loaded polymersomes and OmpF-SH equipped polymersomes 1 hour after addition of 30mM GSH was verified by the two sample t-test ($p < 0.02$, n=3).

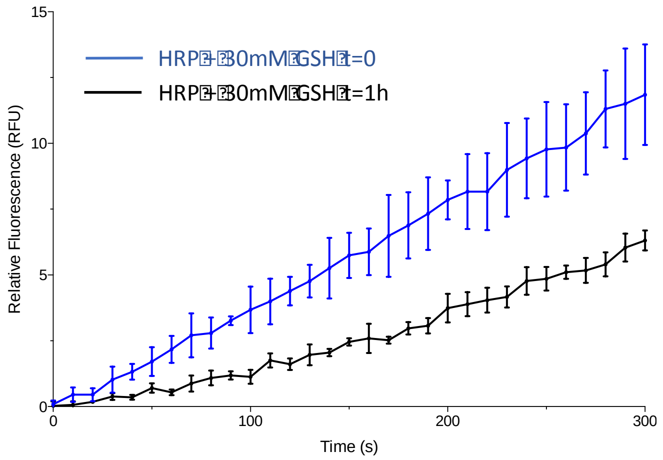

Supplementary Figure 8. Amplex UltraRed conversion of free HRP in the presence of 30mM GSH. Fluorescence intensity immediately after mixing with 30mM GSH in PBS at pH 7.4 (blue), and after 1 hour (black). Error bars represent standard deviations of 3 measurements.

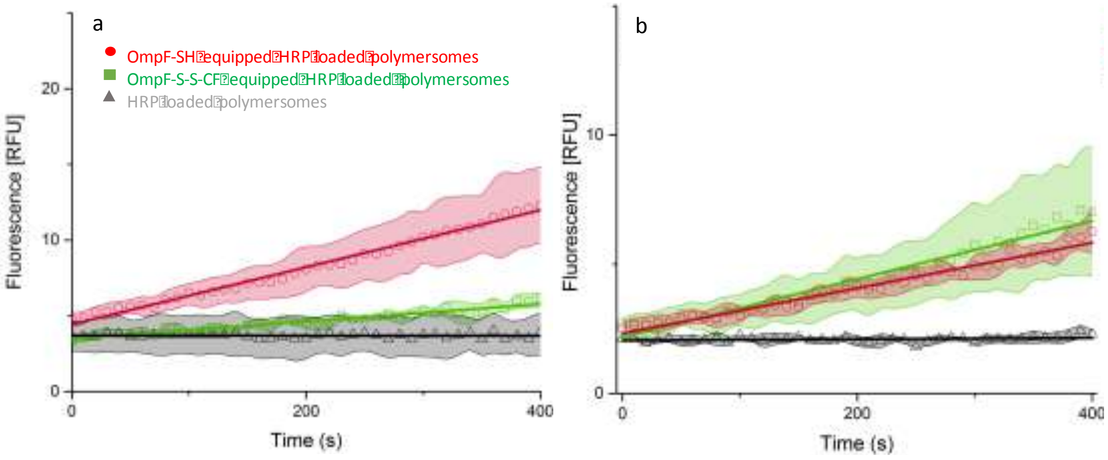

Supplementary Figure 9. **Enzymatic Amplex UltraRed conversion by catalytic nanocompartments.** Fluorescence intensity of OmpF-SH permeabilised catalytic nanocompartments (red), OmpF-S-S-CF permeabelised catalytic nanocompartments (green), unpermeabilised HRP-loaded polymersomes (black) in the presence of 30mM GSH in PBS at pH 7.4 at a.) t=0, and b.) at t=1h. Error bars show standard deviations of 3 measurements.

Cytotoxicity

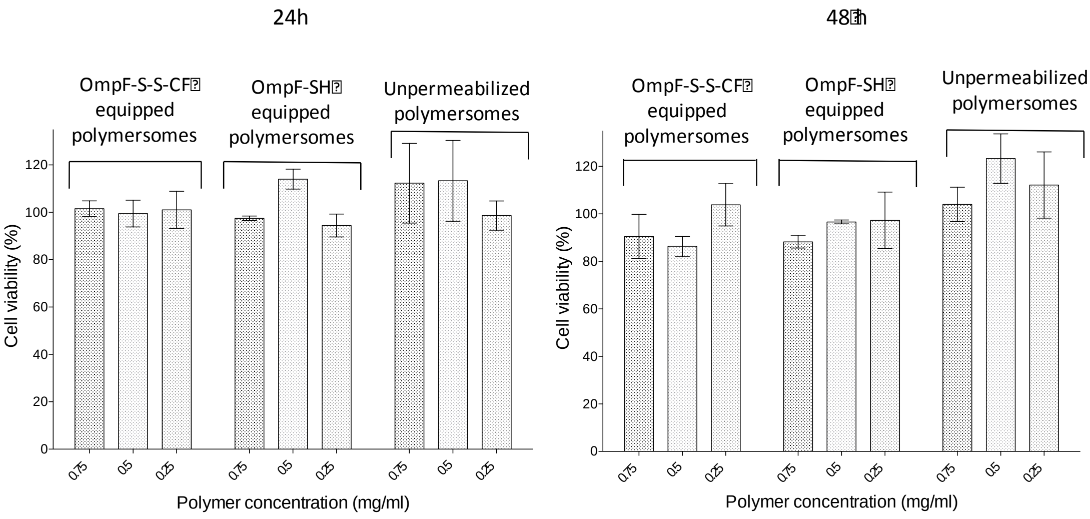

Supplementary Figure 10. **Cytotoxicity of catalytic nanocompartments.** Viability of HeLa cells incubated with HRP-loaded polymersomes, HRP-loaded polymersomes equipped with OmpF-SH and HRP-loaded polymersomes equipped with OmpF-S-S-CF after 24h (left) and 48h (right). Error bars show standard deviations of HeLa cell viability incubated with catalytic nanocomparments for 3 separate measurements.

HRP Conjugation with Atto-488 and Atto-647

For conjugation with Atto-488 carboxylic acid succinimidyl ester (Atto-488 NHS ester) (Atto-TEC, Germany), 0.2 mg ml^{-1} HRP solution was prepared in 0.2 M sodium

bicarbonate buffer pH = 8.1 and 10µL of 10mM Atto-488 NHS ester was added per 1 mL of enzyme solution. The final solution was mixed in the dark for 24h. After labelling, the Atto-488 HRP conjugate was purified by dialysis against PBS at pH 7.4 for 72 hours, exchanging the buffer 3 times daily using 14kDa Membra-Cel™ (Carl Roth, Germany) dialysis membranes. Samples were further purified using Amicon Ultra-15mL Centrifugal Filters for DNA and protein concentration (MWCO: 10kDa) (EMD Millipore, USA). Labelling was confirmed by SDS-PAGE (Supplementary Figure 11 A).

For conjugation with Atto-647 carboxylic acid succinimidyl ester (Atto 647 NHS ester) (Atto-TEC, Germany),0.2 mg ml^{-1} HRP solution was prepared in 0.2 M sodium bicarbonate buffer (pH = 8.1), and 10 µL of 10 mM Atto 647 NHS ester was added per 1 mL of enzyme solution. The final solution was mixed in the dark for 24h. The Atto-488 HRP conjugate was purified as described above for the Atto-488 HRP conjugate and labeling was confirmed by SDS-PAGE (Supplementary Figure 11 B).

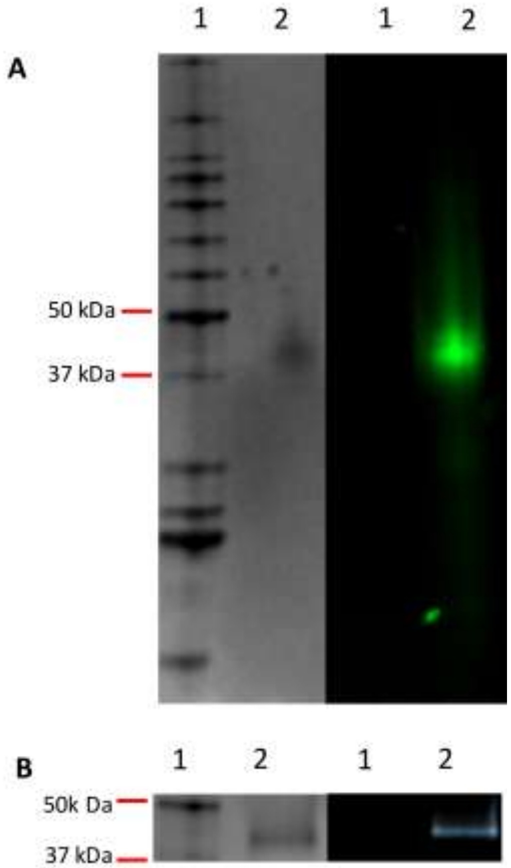

Supplementary Figure 11. 4-20% SDS-PAGE of Atto-488 conjugated HRP and Atto-647 conjugated HRP. A.) Left: Coomassie blue stained gel. Right: Fluorescence image. Lane 1: Protein ladder. Lane 2. Atto-488 Conjugated HRP. B.) Left: Coomassie blue stained gel. Right: Fluorescence image. Lane 1: Protein ladder. Lane 2: Atto-647 conjugated HRP.

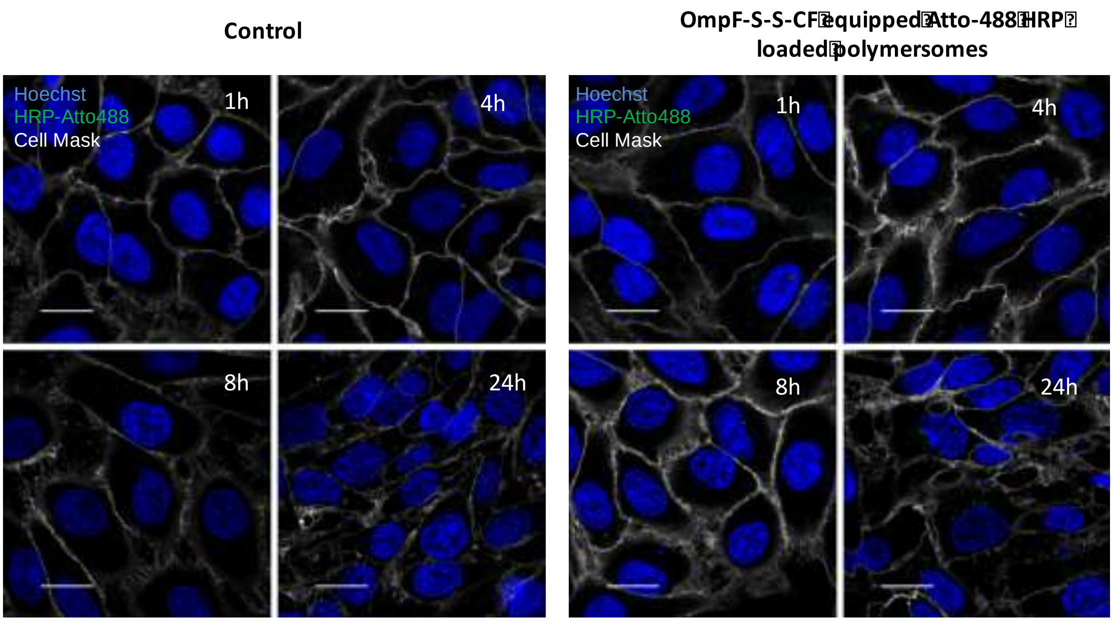

Supplementary Figure 12. Time dependent cellular uptake of Atto-488 conjugated HRP-loaded polymersomes equipped with OmpF-S-S-CF over 24 hours. CLSM micrographs of HeLa cells treated with PBS (Control) or 0.25 mg/ml Atto-488 HRP-loaded polymersomes equipped with OmpF-S-S-CF. Blue: Hoechst 33342 nucleus stain, Gray: CellMask Deep Red Plasma membrane stain, Green: Atto-488. Scale bars: 20 µm

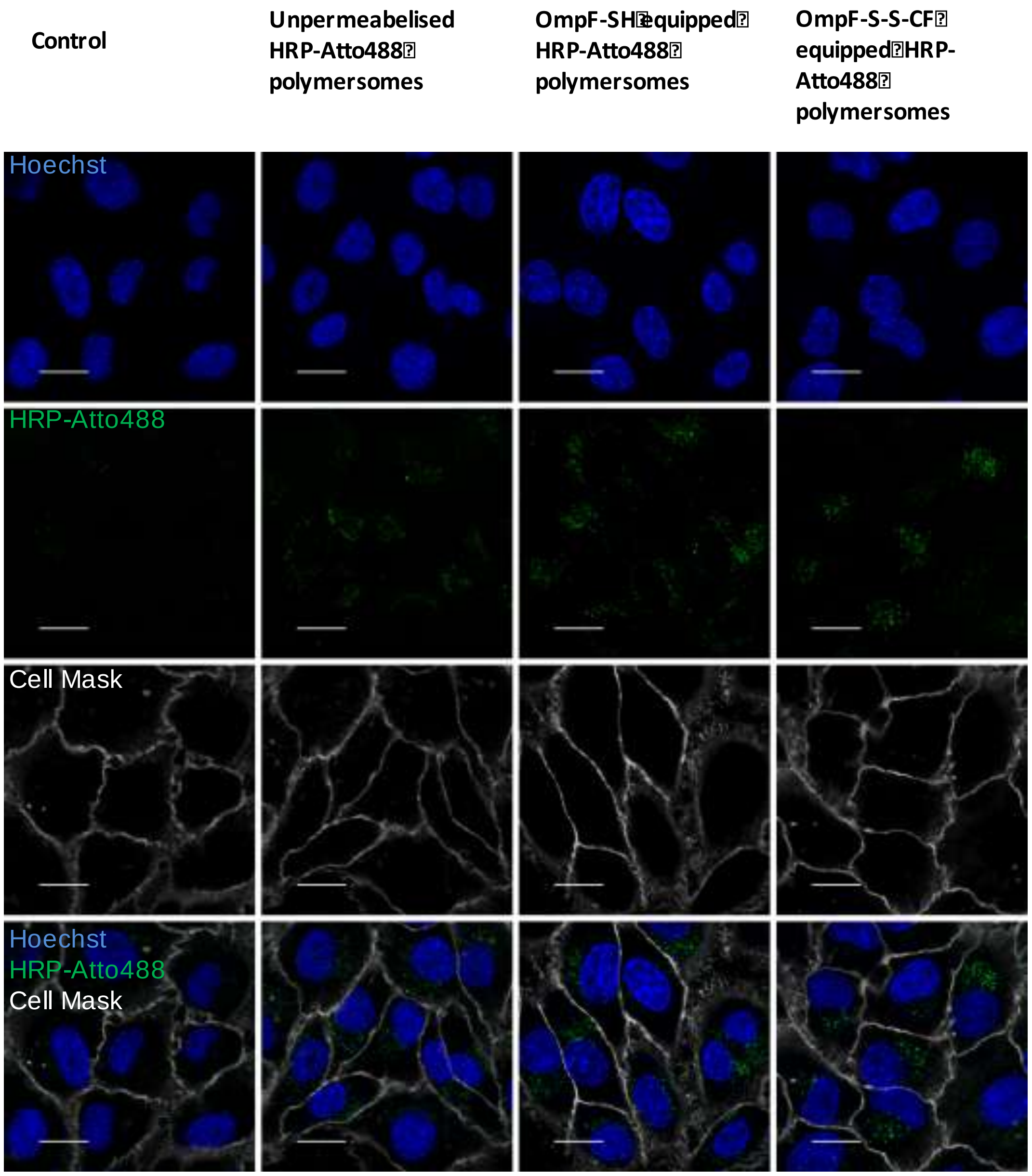

Supplementary Figure 13. Cellular uptake of different HRP-loaded polymersomes after 24 hours. CLSM micrograph of HeLa cells treated with PBS (Control), HRP-Atto488 loaded polymersomes, HRP-Atto488 loaded polymersomes equipped with OmpF-SH and HRP-Atto488 loaded polymersomes equipped with OmpF-S-S-CF (AOs). Blue: Hoechst 33342 nucleus stain, Gray: CellMask Deep Red Plasma membrane stain, Green: Atto-488. Scale bar: 20µm

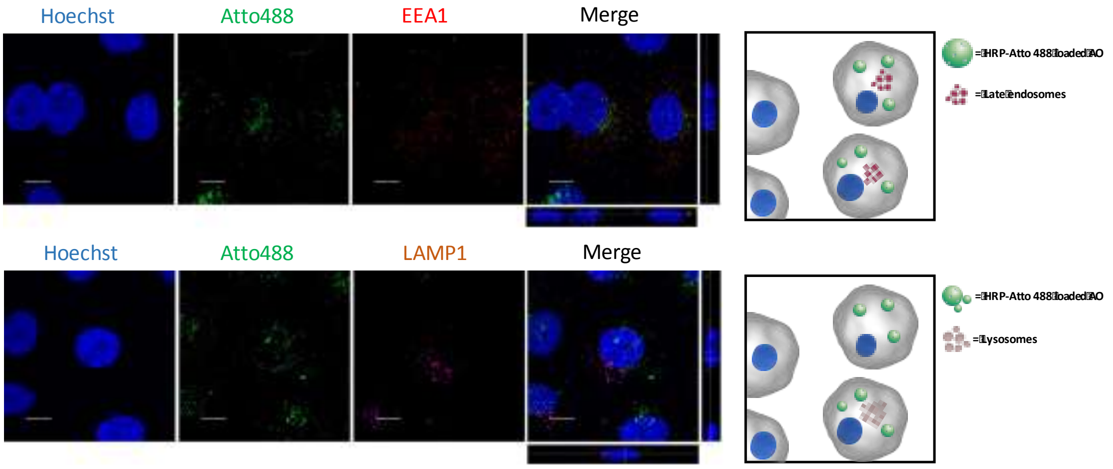

Supplementary Figure 14. Intracellular localization of HRP-loaded polymersomes. Maximum intensity projections including lateral view of HeLa cells treated with HRP-Atto488 loaded polymersomes equipped with OmpF-S-S-CF (AOs) for 24 hours. Endosomes were visualised with EEA1 staining, and lysosomes with LAMP1 staining. Blue: Hoechst 33342 nucleus stain, Green: Atto-488, Red: Early Endosome. Magenta: Lysosome. Scale bar: 10µm

After 24 h AOs (HRP-Atto488 loaded polymersomes equipped with OmpF-S-S-CF) did not co-localize with early endosomes (PCC=0.101 ± 0.028; M1=0.026 ± 0.008, M2=0.003 ± 0.001; Costes=0.047 ± 0.017) or lysosomes (PCC=0.214 ± 0.015; M1=0.029 ± 0.010, M2=0.010 ± 0.005; Costes=0.037 ± 0.008). This confirms successful intracellular endosomal escape.

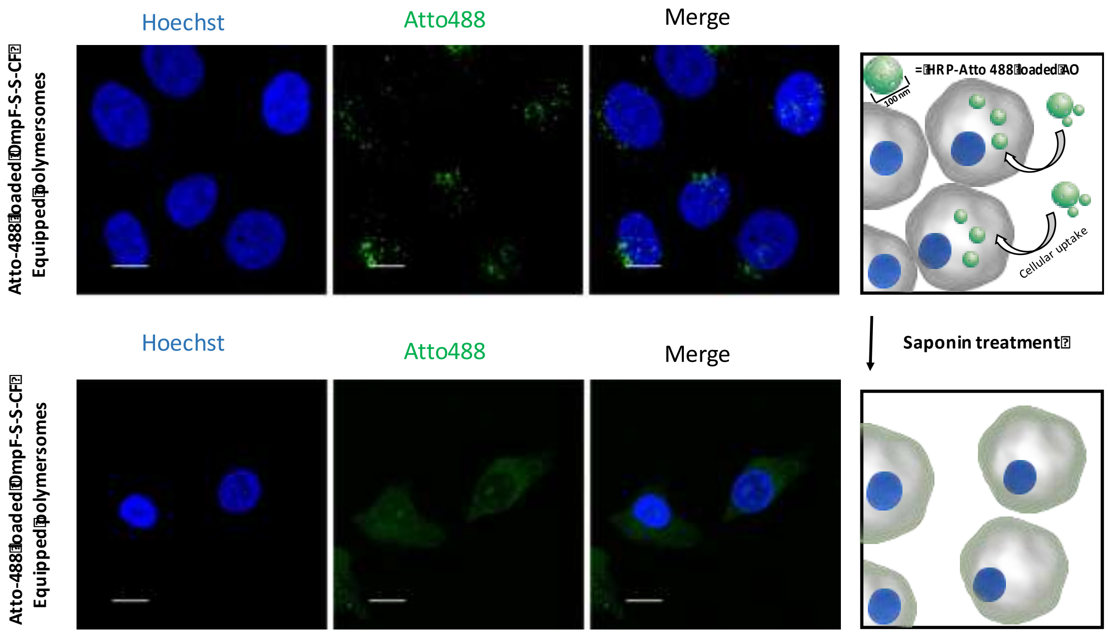

Supplementary Figure 15. Effect of saponin treatment on localization of polymersome encapsulated HRP-Atto488. CLSM maximum intensity projection micrograph of uptake experiments in HeLa cells with AOs (HRP-Atto488 loaded polymersomes equipped with OmpF-S-S-CF). After 24 hours cells were treated with PBS (control) or 0.1% saponin. Blue: Hoechst 33342 nucleus stain, Green: Atto-488. Scale bar: 10µm

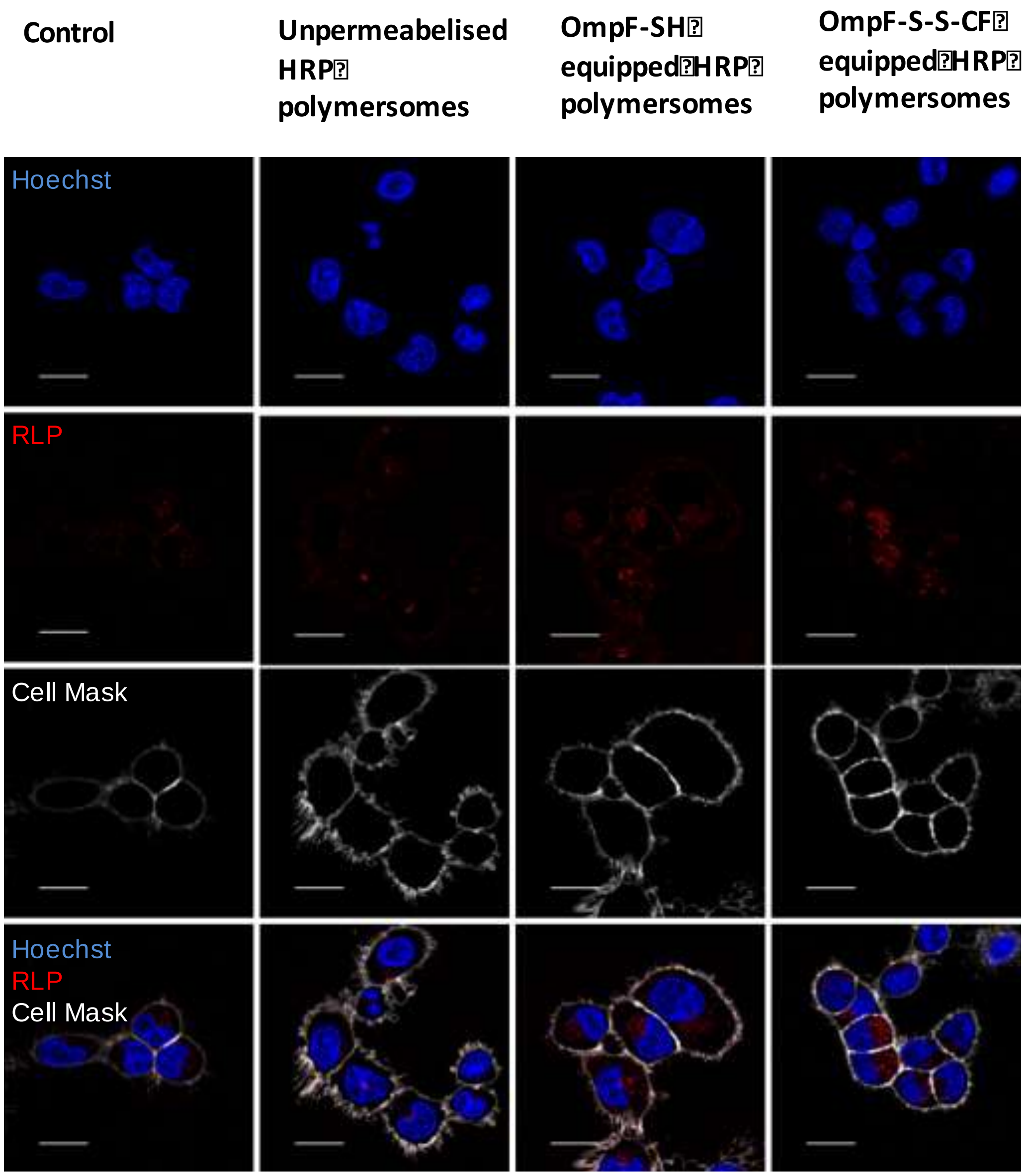

Supplementary Figure 16. Intracellular conversion of Amplex UltraRed by HRP loaded polymersomes and AOs. CLSM micrograph of HeLa cells treated with PBS (Control), unpermeabilised HRP-loaded polymersomes, HRP-loaded polymersomes equipped with OmpF-SH and HRP-loaded polymersomes equipped with OmpF-S-S-CF. Blue: Hoechst 33342 nucleus stain, Cyan: CellMask Deep Red Plasma membrane stain, Red: converted Amplex UltraRed product. Scale bar: 20μm

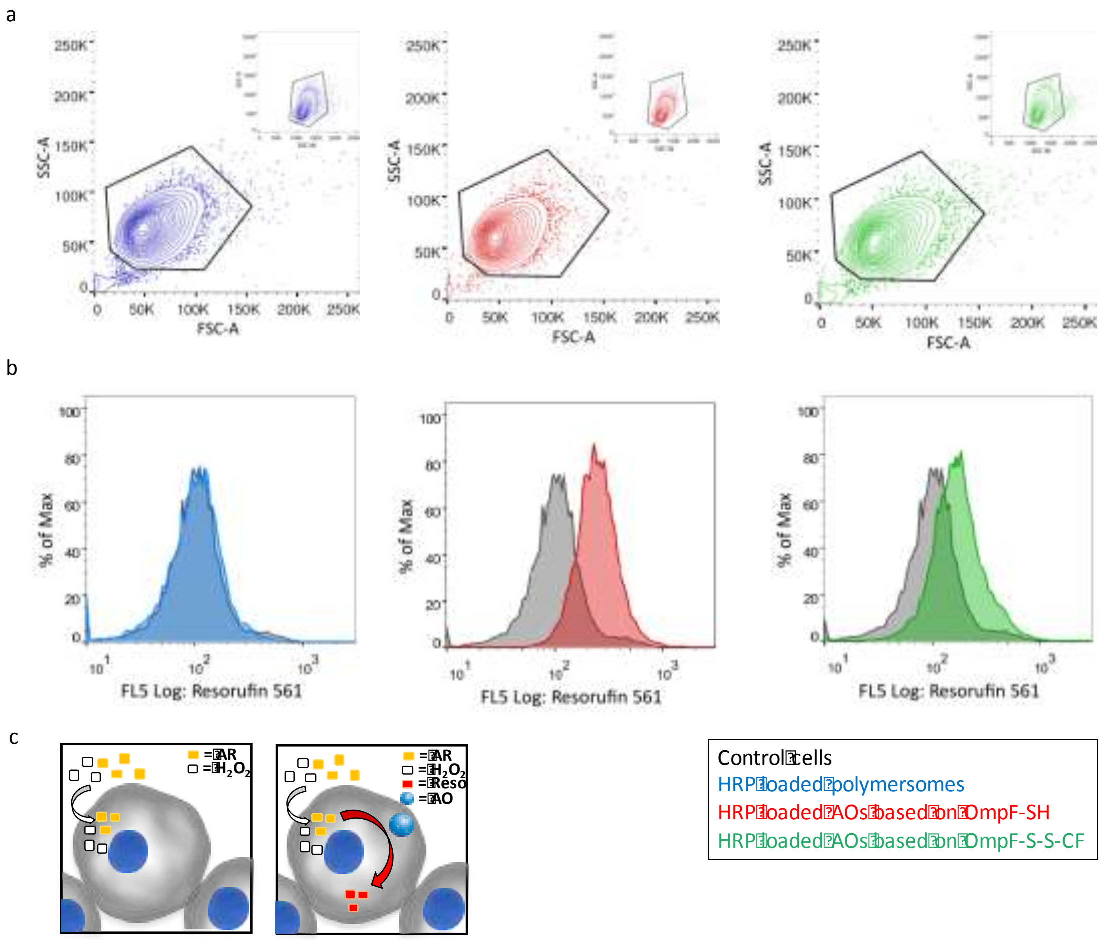

Supplementary Figure 17. Flow cytometry analysis of Amplex Ultra Red Conversion in HeLa cells. Panel a.) Dot plots of FSC area against SSC area are shown. Inserts show the doublet exclusion for the SSC Panel b.) Analysis of Hela cells incubated with: PBS (Gray), HRP loaded polymersomes without OmpF (Blue), and AOs based on: OmpF-SH equipped HRP-loaded polymersomes (Red), OmpF-S-S-CF equipped HRP-loaded polymersome (Green). Panel c.) Shematic representation of the activity of the artificial organelles.

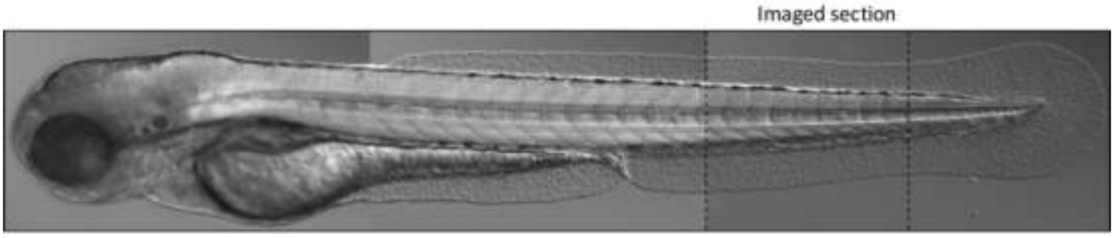

Supplementary Figure 18. Lateral view of the ZFE cross-section used for fluorescence imaging.

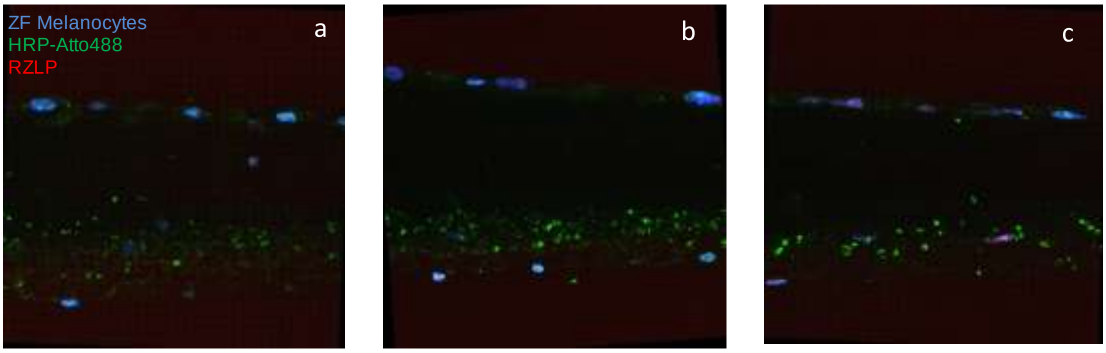

Supplementary Figure 19. Biodistribution of HRP-loaded catalytic nanocompartments in ZFE. Lateral view of the ZFE embryo cross-section. a.) ZFE injected with HRP-loaded polymersomes. b.) ZFE injected with AOs based on HRP-Atto488-loaded polymersomes equipped with OmpF-S-S-CF, c.) ZFE injected with AOs based on HRP-loaded polymersomes equipped with OmpF-SH.

Similar distribution patterns were observed for ZFE injected with unequipped HRP-Atto-488 loaded polymersomes, HRP-Atto-488 loaded polymersomes equipped with OmpF-S-S-CF and those injected with HRP-Atto-488 loaded polymersomes equipped with OmpF-SH. As expected, equipment of polymersomes with OmpF did not have a visible impact on the biodistribution.

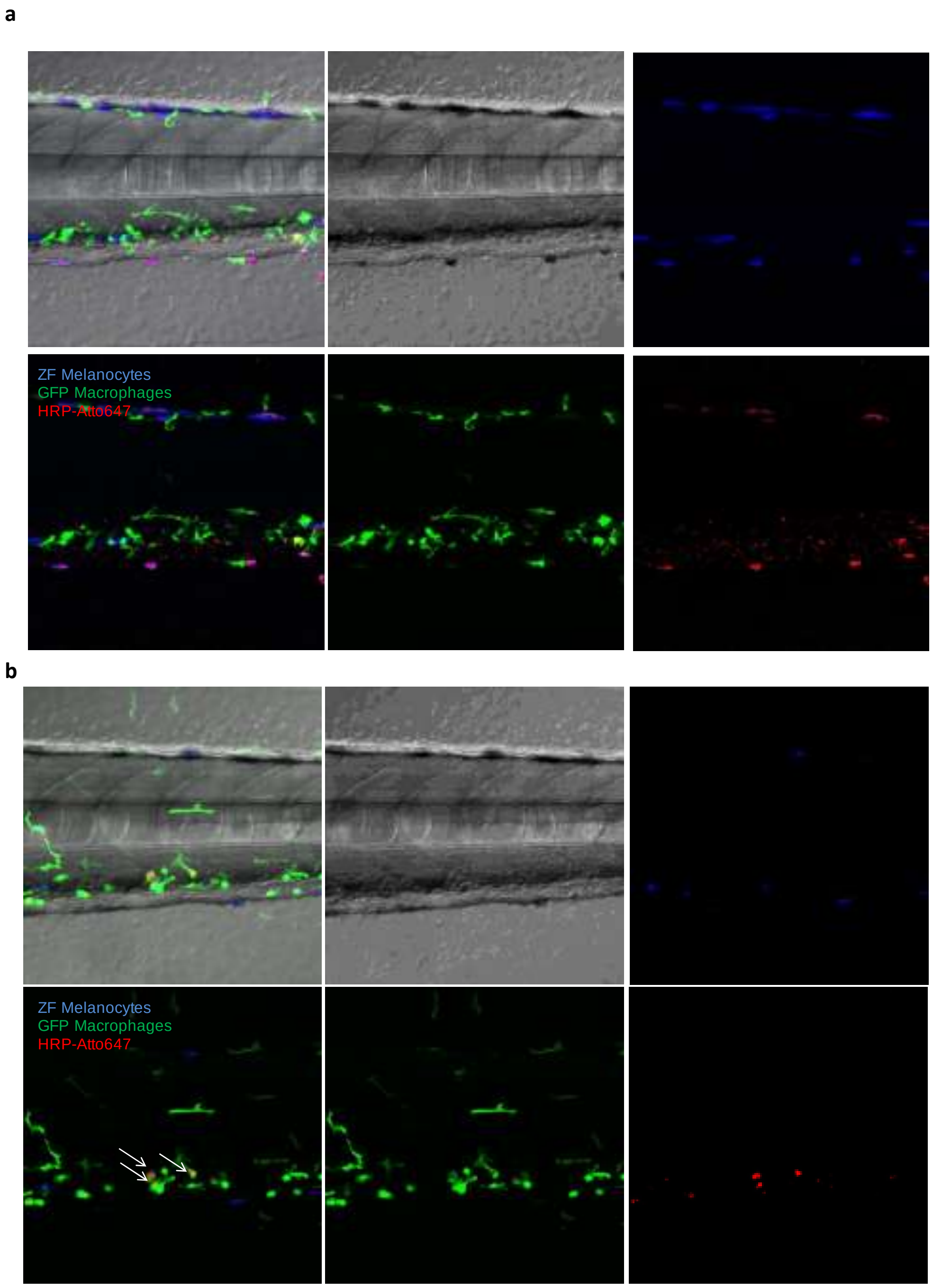

Supplementary Figure 20. Localization of AO in ZFE. Lateral view of the ZFE cross-section. Panel a: ZFE injected with HRP-Atto-647. Panel b: ZFE injected with AOs (HRP-Atto-647 loaded polymersomes equipped with OmpF-S-S-CF). Arrowheads: Localization of AOs.

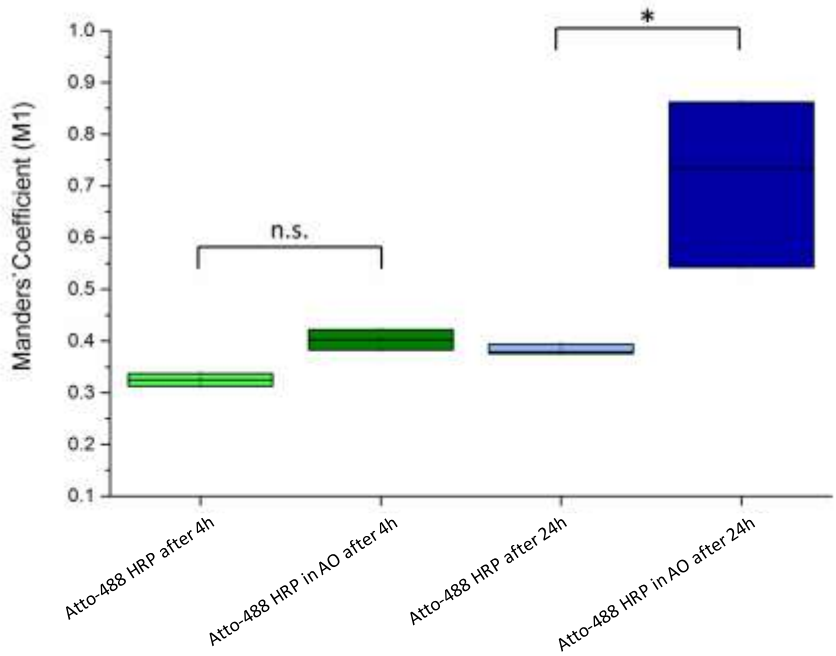

Supplementary Figure 21. Co-localisation of free Atto-488 HRP and Atto-488 HRP-based AOs equipped with OmpF-S-S-CF with zebrafish e-GFP macrophages. Statistical analysis was performed by one-way ANOVA, bonferoni post-hoc test. Colocalisation of free Atto-633 HRP and Atto-633 HRP AO with ZFE e-GFP macrophages. Statistical analysis was performed by one-way ANOVA, bonferoni post-hoc test. Standard deviations represent the data of separately injected ZFE (n = 2 free Atto-633 HRP and n = 3 Atto-633 HRP AO).

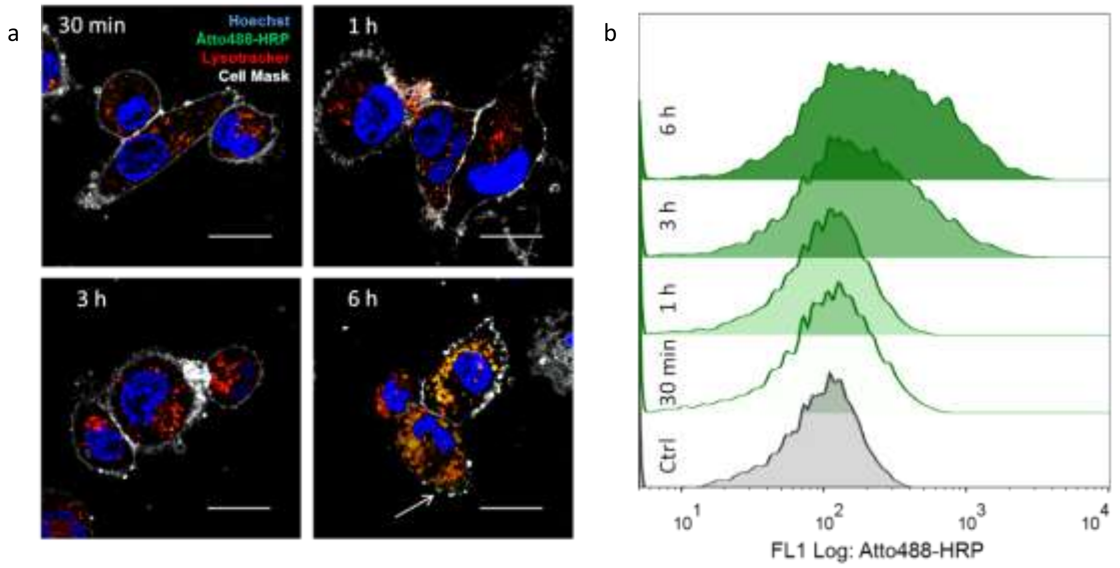

Supplementary Figure 22. Internalization kinetics of AOs by human macrophage differentiated THP-1 cells *in vitro*. Panel a: CLSM micrographs of macrophage differentiated THP-1 cells showing cellular internalization of AOs (Atto488-labelled HRP loaded polymersomes equipped with OmpF-S-S-CF) over time. Blue signal: Hoechst 33342 nucleus stain. Grey signal: CellMask Deep Red-Plasma membrane stain. Green signal: AOs (Atto488-labelled HRP loaded polymersomes equipped with OmpF-S-S-CF). Red signal: LysoTracker™ Red DND-99. Orange signal: Co-localization of AOs and lysosomes. Arrow indicates signal by AOs after lysosomal escape. Scale bar 20µm. Panel b: Quantification of AOs internalization by flow cytometry analysis over time. Control cells (Gray), AOs (Atto488-labelled HRP loaded polymersomes equipped with OmpF-S-S-CF) (Green).

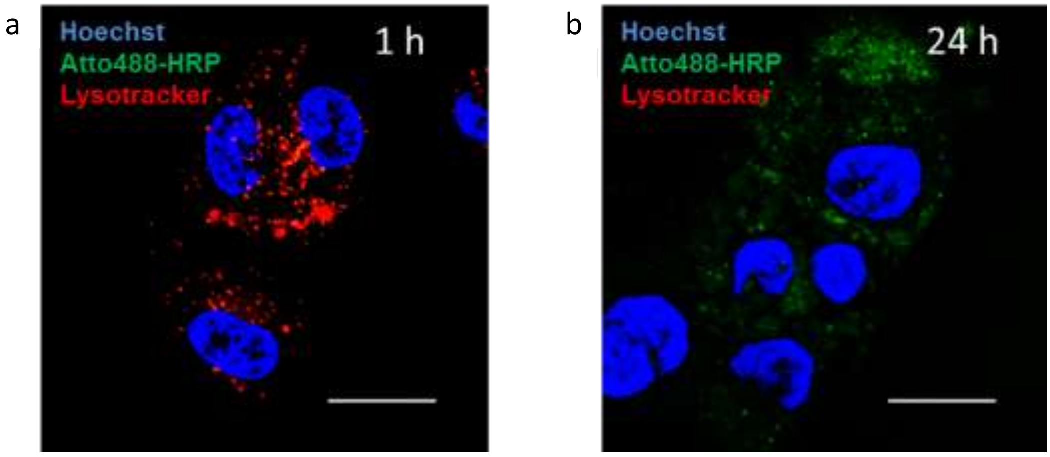

Supplementary Figure 23. Influence of AOs onto lysosomes in human macrophage differentiated THP-1 cells *in vitro*. a. Confocal fluorescence micrographs of macrophage differentiated THP-1 cells incubated for 1 h with AOs (Atto488-labelled HRP loaded polymersomes equipped with OmpF-S-S-CF): strong fluorescence signal of LysoTracker™ Red DND-99. b. Confocal fluorescence micrographs of macrophage differentiated THP-1 cells incubated for 24 h with AOs: no detection of the fluorescence signal of LysoTracker™ Red DND-99. Blue signal: Hoechst 33342 nucleus stain. Green signal: Atto-488 HRP. Red signal: Lysotracker. Scale bar 20µm.

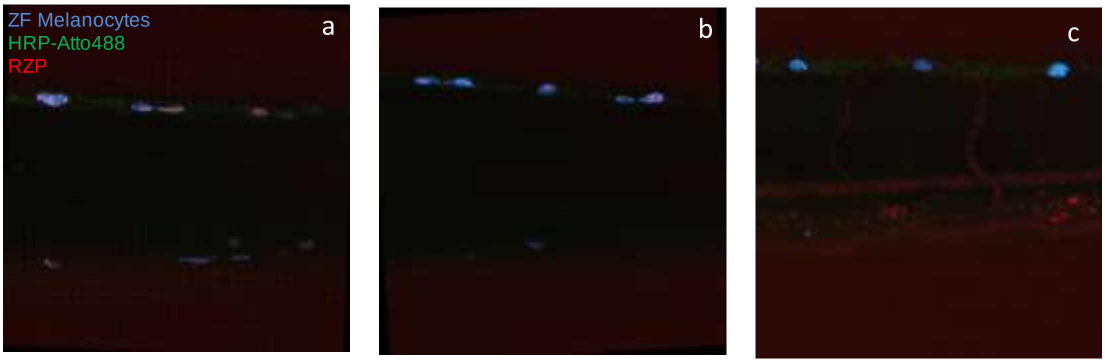

Supplementary Figure 24. Injection of Amplex UltraRed into ZFE Lateral view of the ZFE cross-section. a.) ZFE control. b.) ZFE injected with only Amplex UltraRed, b.) ZFE injected with Amplex UltraRed and H_2O_2.

No resorufin-like product (RLP) or resazurin-like product (RZLP) was observed in the ZFE control or in the ZFE injected with only AR. Injection of AR and H_2O_2 resulted in a staining pattern of the erythrocytes (Supplementary Figure 21).

Supplementary References:

1. Edlinger, C. et al. Biomimetic Strategy to Reversibly trigger functionality of catalytic nanocompartments by the insertion of pH responsive Biovalves. Nano Letters, **17**, 5790-5798 (2017)
2. Itel, F. *et al.* Molecular Organization and Dynamics in Polymersome Membranes: A Lateral Diffusion Study. *Macromolecules* **47,** 7588–7596 (2014).
3. Glatter, T. *et al.* Large-Scale Quantitative Assessment of Different In-Solution Protein Digestion Protocols Reveals Superior Cleavage Efficiency of Tandem Lys-C/Trypsin Proteolysis over Trypsin Digestion. *J. Proteome Res.* **11,** 5145–5156 (2012).
4. Stauch, O., Schubert, R., Savin, G. & Burchard, W. Structure of artificial cytoskeleton containing liposomes in aqueous solution studied by static and dynamic light scattering. *Biomacromolecules* **3,** 565–578 (2002).
5. Güven, A., Fioroni, M., Hauer, B. & Schwaneberg, U. Molecular understanding of sterically controlled compound release through an engineered channel protein (FhuA). *J Nanobiotechnology* **8,** 14 (2010).
6. Sariri, R., Sajedi, R. H. & Jafarian, V. Inhibition of horseradish peroxidase activity by thiol type inhibitors. *Journal of Molecular Liquids* **123,** 20–23 (2006).
